# Study on the correlation of supplementation with *L*-citrulline on the gastrointestinal flora and semen antifreeze performance of ram

**DOI:** 10.3389/fmicb.2024.1396796

**Published:** 2024-05-02

**Authors:** Chen Fan, Aikebaier Aihemaiti, Aoyun Fan, Airixiati Dilixiati, Xi Zhao, Zhuo Li, Changzheng Chen, Guodong Zhao

**Affiliations:** ^1^College of Animal Science, Xinjiang Key Laboratory of Herbivore Nutrition for Meat & Milk, Xinjiang Agricultural University, Urumqi, China; ^2^College of Animal Science, Xinjiang Agricultural University, Urumqi, China

**Keywords:** *L*-Cit, Turpan black sheep, semen, freezing resistance, testis, protein, microflora

## Abstract

**Introduction:**

Cryopreservation of semen can give full play to the reproductive advantages of male animals. However, in actual production, due to the poor frost resistance of sheep semen and the low conception rate, the promotion of sheep frozen semen is greatly hindered. Therefore, it is urgent to improve the frost resistance of semen to improve the quality of frozen semen. At present, most studies on improving the quality of frozen semen are based on the improvement of semen dilutions, and few studies on improving the freezing resistance of ram semen by feeding functional amino acids.

**Methods:**

Therefore, 24 Turpan black rams were divided into high antifreeze group (HF) and a low antifreeze group (LF) Each of these groups was further randomly divided into control and experimental subgroups. The control subgroup was fed a basal diet, while the experimental subgroup received an additional 12 g/d of *L*-Cit supplementation based on the control group for a duration of 90 days.

**Results:**

The results showed that Following *L*-Cit supplementation, the experimental group demonstrated significantly elevated sperm density and VSL (Velocity of straight line), T-AOC, GSH-Px, and NO levels in fresh semen compared to the control group (*P* < 0.01). After thawing, the experimental group exhibited significantly higher levels of T-AOC, GSH-Px, and NO compared to the control group (*P* < 0.01). Additionally, the HFT group, after thawing frozen semen, displayed significantly higher HK1 protein expression compared to the control group. The number of spermatogonia, spermatocytes, and sperm cells in the HFT group was significantly higher than that in the HFC group. Moreover, 16S rRNA sequence analysis showed that *Candidatus_Saccharimonas, Staphylococcus, Weissella, succinivbrionaceae_UcG_002*, and *Quinella* were significantly enriched in the rumen of the HFT group, while *Ureaplasma* was significantly enriched in the HFC group. In the duodenum, *Clostridiales_bacterium_Firm_14, Butyrivibrio*, and *Prevotellaceae_NK3831_group* were significantly enriched in the HFT group, whereas *Desulfovibrio* and *Quinella* were significantly enriched in the HFC group.

**Discussion:**

Under the conditions employed in this study, *L*-Cit supplementation was found to enhance the intestinal flora composition in rams, thereby improving semen quality, enhancing the antifreeze performance of semen, and promoting the development of testicular spermatogenic cells.

## 1 Introduction

Livestock semen cryopreservation holds significant importance in livestock production. During semen cryopreservation, sperm are subjected to cold shock, ice crystal formation, oxidative stress, and other molecular alterations. These factors contribute to the freezing-induced damage, affecting sperm motility, vitality, and structural integrity (Peris-Frau et al., 2020). In the course of freezing livestock semen, aside from the inherent factors associated with the freezing methodology and the technology used to improve sperm quality post-thawing, there are notable variations in semen cryopreservation outcomes among different species due to varying sperm tolerance to low temperatures. Studies have shown that bovine frozen semen typically exhibits approximately 50% sperm motility and viability after thawing (Khalil et al., 2017). Compared with that of cattle, frozen-thawed semen of pigs typically demonstrates approximately 40% sperm motility (Roca et al., 2006). Furthermore, compared with that of cattle and pigs, frozen-thawed semen of sheep displays lower sperm motility and survival rates, at approximately 40% and 30%, respectively (García et al., 2017).

The study of sheep semen cryopreservation technology dates back to the 1950s (Emmens and Blackshaw, 1950). At present, the primary approach to mitigating sperm cryopreservation damage in sheep involves the addition of antioxidants, oligosaccharides, and natural or synthetic ice crystal inhibitor antifreeze protectants to the freezing diluent (Ma et al., 2019). There is limited research on improving semen cryopreservation outcomes through the supplementation of exogenous additives. Recent studies have revealed a close connection between gastric and intestinal flora and semen quality. An imbalance in gastric and intestinal flora can adversely impact spermatogenesis, leading to a reduction in semen quality and male fertility (Ding et al., 2020). Volatile fatty acids (VFAs) absorbed in the rumen play a vital role in providing energy to the animal body. Some VFAs produced during rumen fermentation are absorbed by the rumen epithelium, neutralized by saliva, or absorbed into the small intestine along with chyme (Bergman, 1990). An increase in VFA concentration can lead to a decrease in rumen fluid pH, potentially altering rumen microflora and the type of rumen fermentation if pH remains consistently low. In severe cases, it can lead to the disruption of rumen epithelial barrier function, which, in turn, affects the absorption of VFAs by the rumen epithelium (Aschenbach et al., 2011). Intestinal microbiota can significantly mitigate reproductive damage caused by busulfan, thereby enhancing spermatogenesis and semen quality. Alterations in the abundance of the intestinal *Ruminococcaceae_NK4A214_group* can result in decreased bile acid levels, ultimately impairing spermatogenesis and causing damage to spermatogenic cells (Zhang P. et al., 2021). Ding et al. (2020) found that a high-fat diet disrupts the intestinal flora, resulting in an elevated abundance of *Bacteroides* and *Prevotella* in the intestines of normal mice. This alteration reduces the number of spermatocytes and sperm in the seminiferous tubules of normal mice, resulting in decreased sperm quality (Zhang C. et al., 2021). Studies have demonstrated that dietary fiber supplementation can improve the intestinal flora of boars, stimulate the production of short-chain fatty acids, and improve spermatogenesis and semen quality (Lin et al., 2022).

Citrulline belongs to non-essential amino acids, research found that citrulline is not degraded by microorganisms in the rumen (Gilbreath et al., 2019, 2020). Citrulline can promoted vasodilation, muscle synthesis, lipid decomposition, mitochondrial biosynthesis and protein synthesis, citrulline is involved in the urea cycle in the liver, and citrulline-arginine-NO metabolism occurs in macrophages, which plays an important regulatory role in the body 's vasodilation (Allerton et al., 2018). Our previous study demonstrated that *L*-citrulline (*L*-Cit) supplementation significantly improves sperm motility and density, mitochondrial membrane potential, reproductive hormone levels, and the antioxidant capacity of the serum and seminal plasma in rams. Specifically, the supplementation of 12 g/d/ram exerted the most optimal effect (Zhao et al., 2022). Subsequently, our study revealed that *L*-Cit supplementation could regulate protein digestion and absorption pathways in rams, improve serum amino acid metabolism, modulate the expression of testis-related genes, elevate testicular glycolysis/gluconeogenesis pathways, reduce seminal plasma sugar content, and increase seminal plasma amino acid levels (Zhao et al., 2023). Can feeding rams with citrulline at the dose previously studied by our research group improve the semen quality of rams and the microbial structure in the gastrointestinal tract, thereby improving the antifreeze performance of semen? However, there is a lack of relevant research.

As a non-essential amino acid, *L*-Cit is finally decomposed into NO (Nitric Oxide, NO) and polyamines in the body through two major metabolic pathways of arginine and NO metabolism. Among them, the regulation of *L*-Cit on the reproductive performance of the body mainly depends on the NO cycle. Studies have confirmed that nitric oxide (NO) has multiple roles in organisms. On the one hand, it can effectively reduce the level of androstenedione; on the other hand, it can also regulate the production of testicular steroids (Lundberg and Govoni, 2004). NO not only has the ability to regulate the synthesis of luteinizing hormone releasing hormone (LHRH), but also induces the release and synthesis of prostaglandin E_2_ (PGE_2_) in the hypothalamus. As an important precursor of arginine, *L*-Cit can be synthesized in the intestine of adult animals, and then transported to the kidney to further convert to arginine to meet the body 's normal demand for arginine. The study found that adding 2.33 g/kg *L*-arginine (*L*-Arg) to the cock's diet can effectively promote the increase of cock 's testicular weight, sperm density, the number of straight-line sperm, and the concentration of testosterone in plasma (El-Hattab et al., 2012). However, there is no systematic study on the effect of supplementary feeding *L*-Cit on semen cryopreservation and intestinal flora of rams. Therefore, the purpose of this experiment was to analyze the correlation between semen antifreeze performance and gastrointestinal microorganisms of rams by *L*-Cit supplementation. The purpose of this study was to provide a theoretical basis for the application of *L*-Cit in sheep production.

## 2 Materials and methods

### 2.1 Ethic statement

All animal care and handing procedures in this study were conducted under the guidance of the Care and Use of Laboratory Animals in China and were approved by (protocol number: 2020022) the Animal Care Committee of Xinjiang Agricultural University (Urumqi, Xinjiang, China).

### 2.2 Study locationm

This study was conducted at Huishang Ecological Animal Husbandry Co., Ltd. in Toksun County, Turpan City, Xinjiang (located at coordinates 87°14′05“-89°11′08”E and 41°21′14“-43°18′11”N) over a 90-day period from September to December 2022. The average outdoor and indoor temperatures during this period were 14.2°C and 16.3°C, respectively.

### 2.3 Primary reagents

*L*-Cit (≥98.0% purity, reagent grade) was obtained from Shanghai Shenggong Bioengineering Technology Service Co., Ltd. for refeeding trials. Anhydrous glucose, anhydrous citric acid, and Tris were procured from Shanghai Sigma Company; Sodium penicillin, streptomycin sulfate, and glycerol were obtained from Amresco (USA) were used to prepare semen frozen diluent. Total antioxidant capacity (T-AOC), catalase (CAT), glutathione peroxidase (GSH-Px), and nitric oxide (NO) kits were obtained from Nanjing Jiancheng Bioengineering Institute; A Giemsa staining kit was purchased from Shanghai Sangon Bioengineering Technology Service Co., Ltd were used to determine the antioxidant index, sperm deformity rate and acrosome integrity rate in seminal plasma. Paraffin (melting points: 52°C−54°C, 56°C−60°C, and 60°C−62°C), eosin staining solution, hematoxylin staining solution, neutral gum, and xylene were provided by Tianjin Baishi Chemical Co., Ltd; Ethanol (75%, 80%, 90%, 95%, and 100%), xylene-anhydrous ethanol solution (1:1, v/v), 1% hydrochloric acid ethanol solution, 1% ammonia, and 4% paraformaldehyde were procured from Tianjin Baishi Chemical Co., Ltd.; Paraffin wax was obtained from Leica. Xylene was purchased from Tianjin Baishi Chemical Co., Ltd.; PBS (phosphate buffer saline) (10×), 30% hydrogen peroxide solution, and neutral gum were obtained from Biotopped. H&E (hematoxylin-eosin staining) staining reagents were obtained from Nanchang Yulu Experimental Equipment Co., Ltd. are used for staining and observation of testicular tissue.

### 2.4 Primary equipment

The microplate reader (iMark) was obtained from Bio-Rad (USA) and the centrifuge 5,424 was procured from Eppendorf (Germany) was used to detect the antioxidant index in seminal plasma. The computer-assisted sperm analysis (CASA) system (2001532) was obtained from Auri and was used to detect routine sperm parameters. A semi-automatic rotary slicing machine (German Leica; RM2245) was purchased from Beijing Lebo Liantai Technology Co., Ltd.; A 37°C incubator (model LS-CO150) was purchased from Beijing Wuzhou Oriental Technology Development Co., Ltd.; A tissue embedding machine (model BMJ-1) was procured from Tianjin Tianli Aviation Electromechanical Co., Ltd.; A biological tissue staining machine (model RS-18II) was purchased from Hubei Xiaogan Kuohongye Medical Instrument Co., Ltd.; The German SLEE tissue dehydrator (model MTP) and German SLEE baking sheet machine (model Slidetec WATER/HETA) were purchased from Beijing Lybyshin Technology Development Co., Ltd.; An optical microscope (model L2800) was purchased from Shenzhen Xingming Optical Instrument Co., Ltd.; The Merck Millipore pure water meter and toasting machine were provided by Leica; We also obtained a pH meter (OHAUS^®^), a biological microscope (Nikon), Bouin's solution (Beijing Regen Biotechnology Co., Ltd.), and a water bath all used for tissue section preparation and observation.

### 2.5 Feed management

All test rams were fed under the same feeding conditions, receiving meals at 09:30 and 17:30 daily via a TMR (Total Mixed Ration) feeding vehicle with unrestricted access to both food and water. The dietary composition and nutritional content of the rams are detailed in Table 1.

**Table 1:** TMR composition and nutritional levels (based on dry matter, %).

**Diet composition**	**Proportion**	**Nutritional level**	**Content**
Alfalfa Hay	24.25	Organic matter	89.85
Wheat straw hay	33.61	Crude protein, %	10.38
Hay	26.50	Neutral detergent fiber, %	47.87
Concentrate supplements	13.31	Acid detergent fiber, %	31.96
baking soda	1.20	Ca, %	1.31
Nacl	1.04	P, %	0.19
Total	100.00	ME, MJ/kg	9.75

The nutrient content represents the measured value, while metabolic energy denotes the calculated value.

### 2.6 Study design

Twenty-four Turpan black sheep rams, characterized by symmetrical body conditions, good semen quality, and an average body weight of 65.24 ± 5.41 kg, were selected for pseudovaginal sperm collection. Subsequently, 50 μL of fresh semen was obtained and diluted 60-fold with a diluent. A 5 μL aliquot was then placed on a slide, covered with a glass slide, and subjected to analysis using the CASA system. At least five fields and 1,000 sperm cells were examined. The fresh sperm motility rate exceeded 85%, and the semen samples with sperms exhibiting rapid linear motion of more than 70% were subjected to freezing.

The qualified semen was diluted to a concentration of 5.0 × 107 sperm/mL at a room temperature of 25°C, and promptly sub-packed into 0.25 mL Kasu frozen semen tubes. Subsequently, the tubes were sealed, wrapped in 16 layers of gauze, and placed in a refrigerator at 4°C for a 3-h cooling and balancing period. Afterwards, the samples were evenly spaced on a freezing rack, fumigated with liquid nitrogen at a distance of 3.5 cm from the liquid nitrogen level for 8 min, and promptly transferred into liquid nitrogen and sub-packed into finger tubes for storage. On the third day post-freezing, the frozen semen was thawed in a 37°C water bath for 30 s. Based on post-thaw viability, 24 rams were sorted by descending sperm viability, with 12 rams exhibiting sperm viability exceeding 50% designated as the high antifreeze group (HF), while the remaining 12 rams with sperm viability below 50% were categorized as the low antifreeze group (LF). The 12 sheep in the HF group were randomly allocated into control (HFC) and experimental (HFT) subgroups, each consisting of 6 sheep. Similarly, the 12 sheep in the LF group were randomly assigned to the control (HFC) and experimental (HFT) subgroups, each comprising 6 sheep. During the experimental period, the rams in the control groups were fed a basal diet. Meanwhile, in the experimental groups, 12 g of *L*-Cit was measured daily at 9 a.m. and placed in a clean mineral water bottle. After completely dissolving the *L*-Cit in 100 mL of tap water, the rams were fixed, and the *L*-Cit solution was administered from the corner of their mouths to ensure complete swallowing.

Frozen diluent: 3.04 g Tris, 1.136 g anhydrous glucose, 1.554 g anhydrous citric acid, and 1,000 IU (International Unit) of antibiotics were dissolved in deionized water and subsequently diluted to a final volume of 100 mL. Following filtration through a 0.22-mm filter, 10% egg yolk and 6% glycerol were incorporated.

### 2.7 Semen collection

The semen was collected using a pseudovagina every 5 days, with two collections occurring at an 8-min interval. Semen discharge took place once daily, preceding each semen collection. Semen samples from rams on both days 0 and 90 were collected for experimentation.

### 2.8 Collection of testicular samples, rumen fluid, and duodenal contents

On the 90th day of the experiment, the abdominal cavities of the rams in both the HFC and HFT groups were promptly opened following slaughter. After removing the rumen, it was incised, and the intermediary contents were wrapped with five layers of gauze. The rumen fluid was squeezed and collected into cryopreservation tubes, which were subsequently stored in liquid nitrogen. Simultaneously, the midsection of the duodenum was obtained, and its contents were squeezed out into frozen tubes and stored in liquid nitrogen. Simultaneously, the left testicular tissue from each ram was collected and stored in 4% paraformaldehyde for histological analysis.

### 2.9 Detection of sperm routine parameters

CASA was employed to assess sperm density, total motility (TM), progressive motility (PM), and motility rate. Fresh semen was diluted by a factor of 100 using a diluent (10 μL of semen + 990 μL of diluent). The ruby counting plate was positioned on the sperm analyzer's temperature-controlled stage, maintained at 37°C. After preheating, 10 μL of absorbed sperm droplets were placed onto the counting plate. Five fields of view were observed for each sample, and the system automatically quantified sperm density, TM, PM, as well as various motion parameters such as VCL (Velocity of cured line), VSL(Velocity of straight line), VAP (Velocity average path), and others.

The sperm deformity rate was assessed through Giemsa staining (Zhou et al., 2021). The sperm plasma membrane integrity rate was evaluated using the hypo-osmotic swelling test method (Zakošek et al., 2020). Sperm acrosome integrity rate was determined via Giemsa staining (Serafini et al., 2014). The measurement of sperm antioxidant indexes T-AOC, CAT, GSH-Px, and NO followed kit instructions and was performed using a microplate reader.

### 2.10 Protein quantification

Three rams from both the HFC and HFT groups were randomly selected and observed for a period of 90 days to assess the expression of HSP90 and hexokinase 1 (HK1) proteins in thawed semen (Zhang C. et al., 2021).

### 2.11 Testicular tissue structure analysis

On the 90th day of the experiment, the rams from both the HFC and HFT groups were euthanized, and their left testes were excised. After making an incision, a 2 × 2 cm section of testicular tissue from the same region was obtained and subsequently transformed into a tissue block measuring approximately 10 mm × 10 mm × 5 mm. These sections were stained, dried, sealed with a neutral gum sealing agent, and microscopically examined (Zhou et al., 2021).

### 2.12 Microflora sequencing and data analysis of rumen fluid and duodenal contents samples

(I) Preparative genomic DNA extraction and PCR amplification. The genomic DNA of the samples was extracted by sodium dodecyl sulfonate (SDS) method. The diluted genomic DNA was used as template, 16S V3-V4 was selected as sequencing region, and PCR amplification was performed using specific primers and high fidelity enzymes.

(II) Library preparation and sequencing. The V3-V4 region of the 16S rRNA gene was amplified using the primers MPRK341F (59-ACTCCTACGGGAGGC AGCAG-39) and MPRK806R (59-GGACTACHVGGG TWTCTAAT-39) with barcoding. The PCRs (total, 30 mL) included 15 mL Phusion high-fidelity PCR master mix (New England Biolabs), 0.2 mM primers, and 10 ng DNA. The thermal cycle was carried out with an initial denaturation at 98°C, followed by 30 cycles of 98°C for 10 s, 50°C for 30 s, 72°C for 30 s, and a final extension at 72°C for 5 min. PCR products were purified using a GeneJet gel extraction kit (Thermo Scientific, USA). The sequencing libraries were constructed with NEBNext Ultra DNA library prep kit for Illumina (NEB, USA) following the manufacturer's instructions, and index codes were added. Then, the library was sequenced on the Illumina HiSeq 2500 platform, and 300-bp paired-end reads were generated at the *Novo* gene. The paired-end reads were merged using FLASH (v.1.2.71). The quality of the tags was controlled in QIIME (v.1.7.02); meanwhile, all chimeras were removed. The “core set” of the Greengenes database was used for classification, and sequences with 0.97% similarity were assigned to the same operational taxonomic units (OTUs).

(III) Analysis of sequencing data. Operational taxonomic unit abundance information was normalized using a standard sequence number corresponding to the sample with the least sequences. The alpha diversity index was calculated with QIIME (v.1.7.0). The UniFrac distance was obtained using QIIME (b1.7.0), and principal-coordinate analysis (PCA) was performed using R software (v.2.15.3). The linear discriminate analysis effect size (LEfSe) was performed to determine differences in abundance; the threshold LDA score was 4.0. GraphPad Prism9.0 software and origin software were used to make charts and correlation analysis (Yan et al., 2022).

### 2.13 Statistical analysis

Microsoft Excel was used to preliminarily sort out the data of semen parameters, spermatogenic cells and related proteins. Subsequently, SPSS 26.0 was used to perform independent sample *t*-test on the data of spermatogenic cells and related proteins. At the same time, the semen indexes were analyzed by one-way analysis of variance, and then Duncan's multiple comparison test was used to evaluate the differences between groups. Origin was used to analyze the correlation between semen parameters and gastric and intestinal flora, and Graph Pad Prism 9.0 and Figdraw were used for data visualization.

## 3 Results

### 3.1 Comparison of semen quality between the high antifreeze group and the low antifreeze group prior to *L*-Cit supplementation

**Figure 1:**
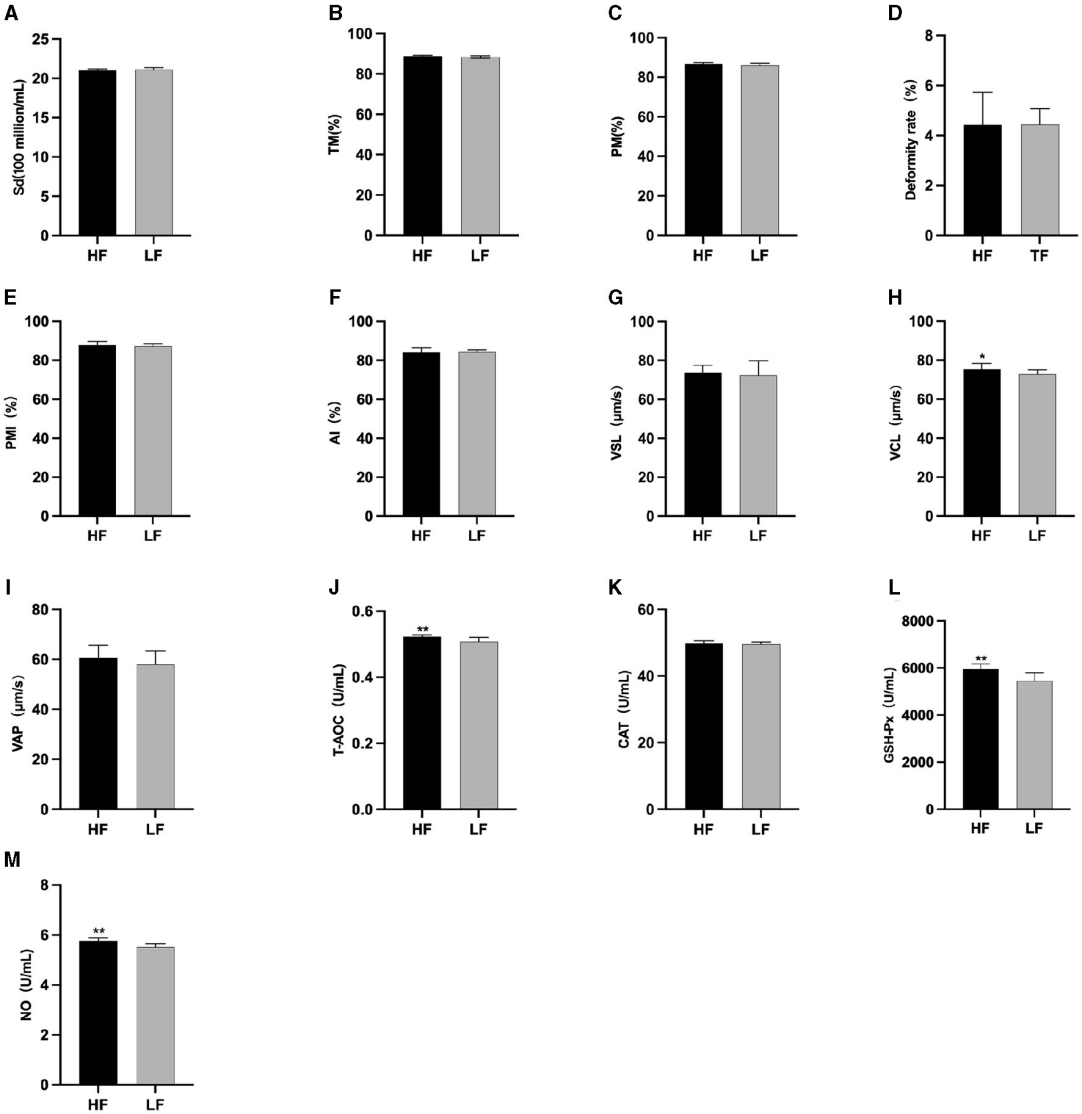
Parameter difference of fresh sperm before supplementation with *L*-Cit. **(A)** Sd, Sperm density; **(B)** TM, Total motility; **(C)** PM, Progressive motility; **(D)** Deformity rate; **(E)** PMI, Plasma membrane integrity, **(F)** AI, Acrosome integrity; **(G)** VSL, Velocity of straight line; **(H)** VCL, Velocity of cured line; **(I)** VAP, Velocity average path; **(J)** T-AOC; **(K)** CAT; **(L)** GSH-Px; **(M)** NO. Compared with the LF group, *P < 0.05; **P < 0.01. Same below.

The semen analysis results prior to freezing are shown in Figure 1. Before *L*-Cit feeding, the sperm curve movement rate in the HF group was significantly higher than that in the LH group (*P* < 0.05) (Figure 1H). Additionally, the levels of T-AOC (Figure 1J), GSH-Px (Figure 1L), and NO (Figure 1M) were significantly elevated in the HF group compared to the LF group (*P* < 0.01).

**Figure 2:**
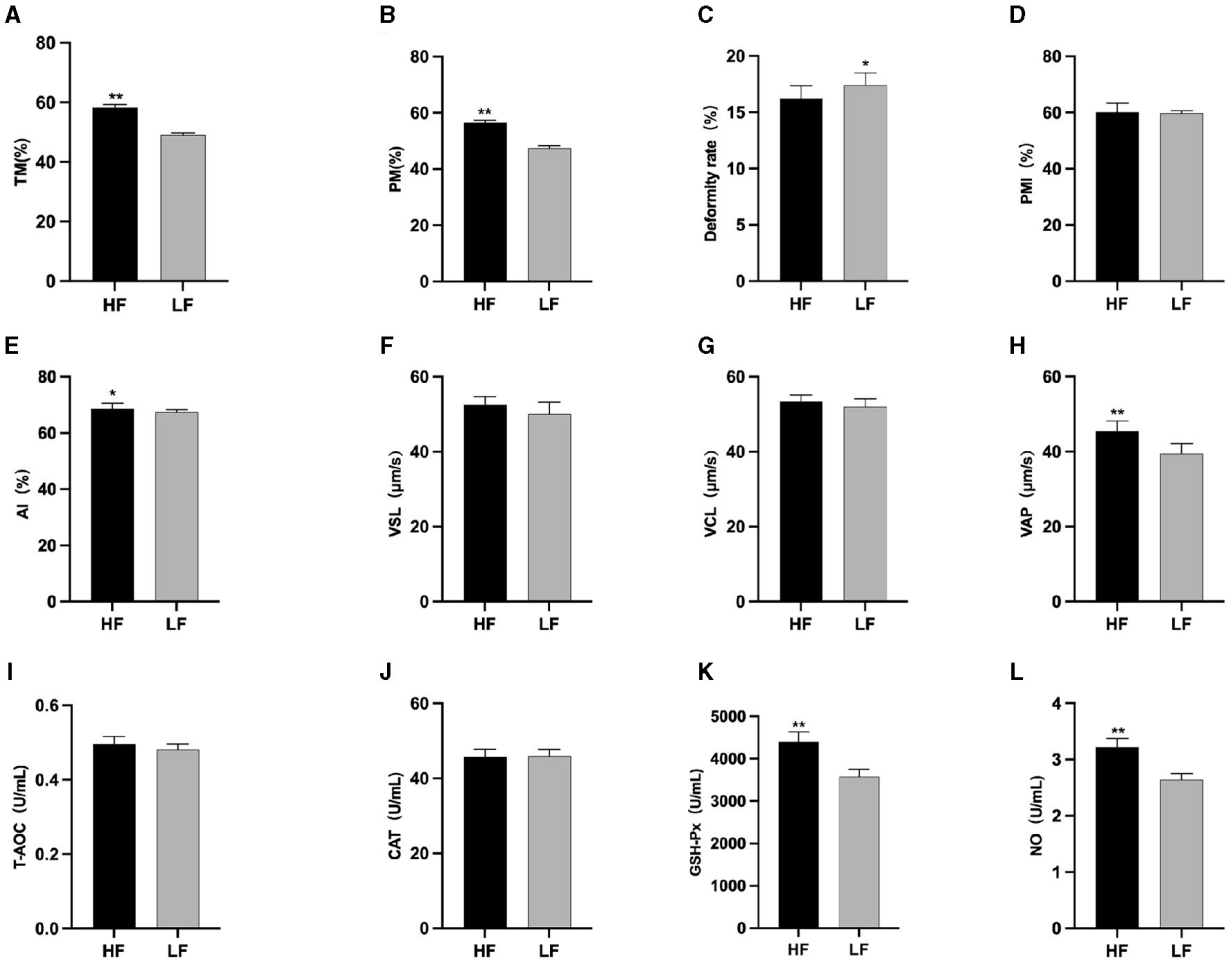
Differences in sperm parameters after thawing before supplementation with *L*-Cit. **(A)** TM, Total motility; **(B)** PM, Progressive motility; **(C)** Deformity rate; **(D)** PMI, Plasma membrane integrity; **(E)** AI, Acrosome integrity; **(F)** VSL, Velocity of straight line; **(G)** VCL, Velocity of cured line; **(H)** VAP, Velocity average path; **(I)** T-AOC; **(J)** CAT; **(K)** GSH-Px; **(L)** NO. Compared with the LF group, *P < 0.05; **P < 0.01.

The results of semen thawing are depicted in Figure 2. The TM (Figure 2A), PM (Figure 2B), VAP (Figure 2H), GSH-Px (Figure 2K), and NO (Figure 2L) in the HF group were significantly higher than those in the LF group (*P* < 0.01). Furthermore, the PMI in the HF group exhibited a significant increase compared to the LF group (*P* < 0.05) (Figure 2E). The LF group displayed a significantly higher sperm deformity rate (Figure 2C) than that in the HF group (*P* < 0.05).

### 3.2 Comparison of fresh and fine quality between the high antifreeze group and the low antifreeze group following *L*-Cit supplementary feeding

**Figure 3:**
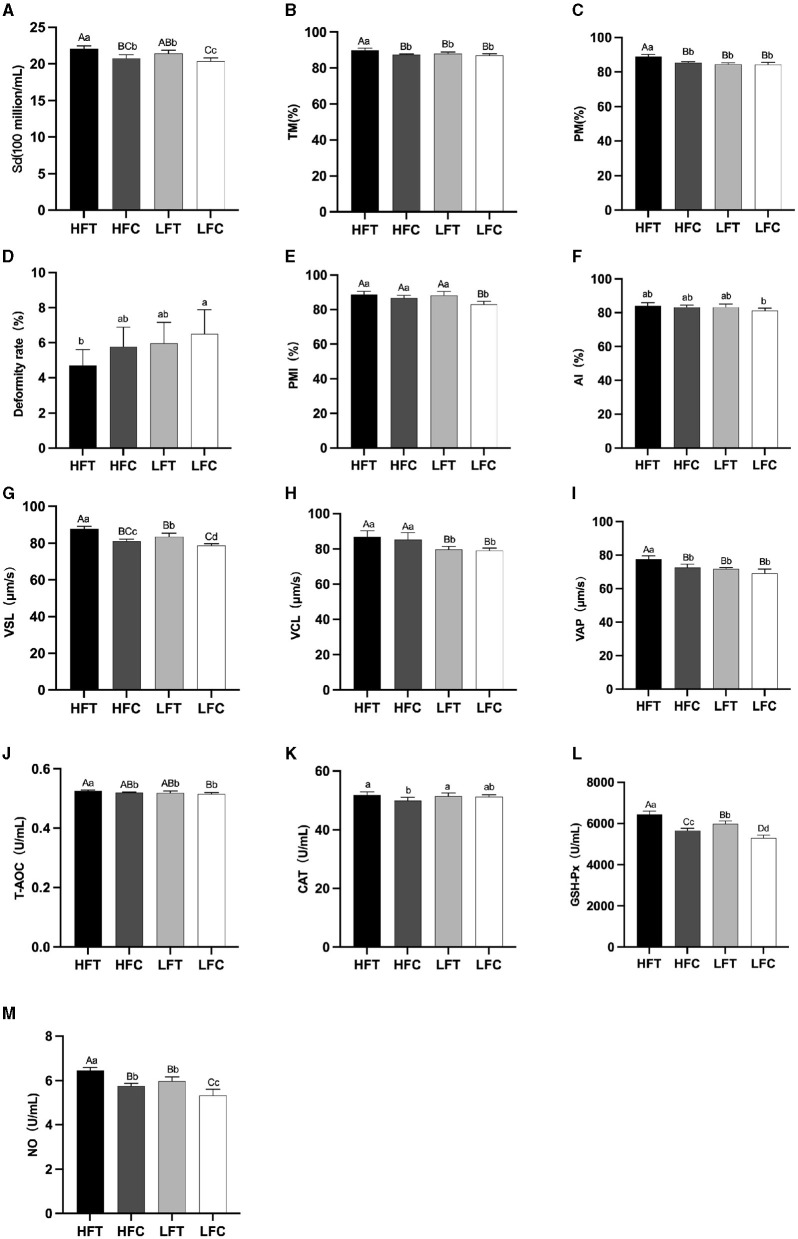
Parameter difference of fresh sperm after supplementation with *L*-Cit. **(A)** Sd, sperm density; **(B)** TM, Total motility; **(C)** PM, Progressive motility; **(D)** Deformity rate; **(E)** PMI, Plasma membrane integrity, **(F)** AI, Acrosome integrity; **(G)** VSL, Velocity of straight line; **(H)** VCL, Velocity of cured line; **(I)** VAP, Velocity average path; **(J)** T-AOC; **(K)** CAT; **(L)** GSH-Px; **(M)** NO. Compared with the LFC group. The difference between different uppercase letters was extremely significant (*P* < 0.01). There was no significant difference between the same letter or no letter label (*P* > 0.05). The same below.

The results of semen analysis prior to freezing are shown in Figure 3. After *L*-Cit supplementation, semen density (Figure 3A) in the LFC group was significantly lower than that in the other three groups (*P* < 0.05). Additionally, sperm acrosome integrity (AI) (Figure 3F) in the LFC group was significantly lower than that of the HFT group (*P* < 0.05). The TM, PM, VSL (Figure 3G), VAP (Figure 3I), T-AOC (Figure 3J), GSH-Px (Figure 3K), and NO (Figure 3L) in the LFT group were significantly higher than those in the other three groups (*P* < 0.01). Furthermore, sperm density (Figure 3A), sperm plasma membrane integrity rate (Figure 3F), linear motion rate (Figure 3G), T-AOC (Figure 3J), GSH-Px (Figure 3K), and NO (Figure 3L) in the low antifreeze test group were significantly higher than those in the low antifreeze control group (*P* < 0.05).

### 3.3 Comparison of semen quality following thawing between the high antifreeze and low antifreeze groups after *L*-Cit supplementary feeding

**Figure 4:**
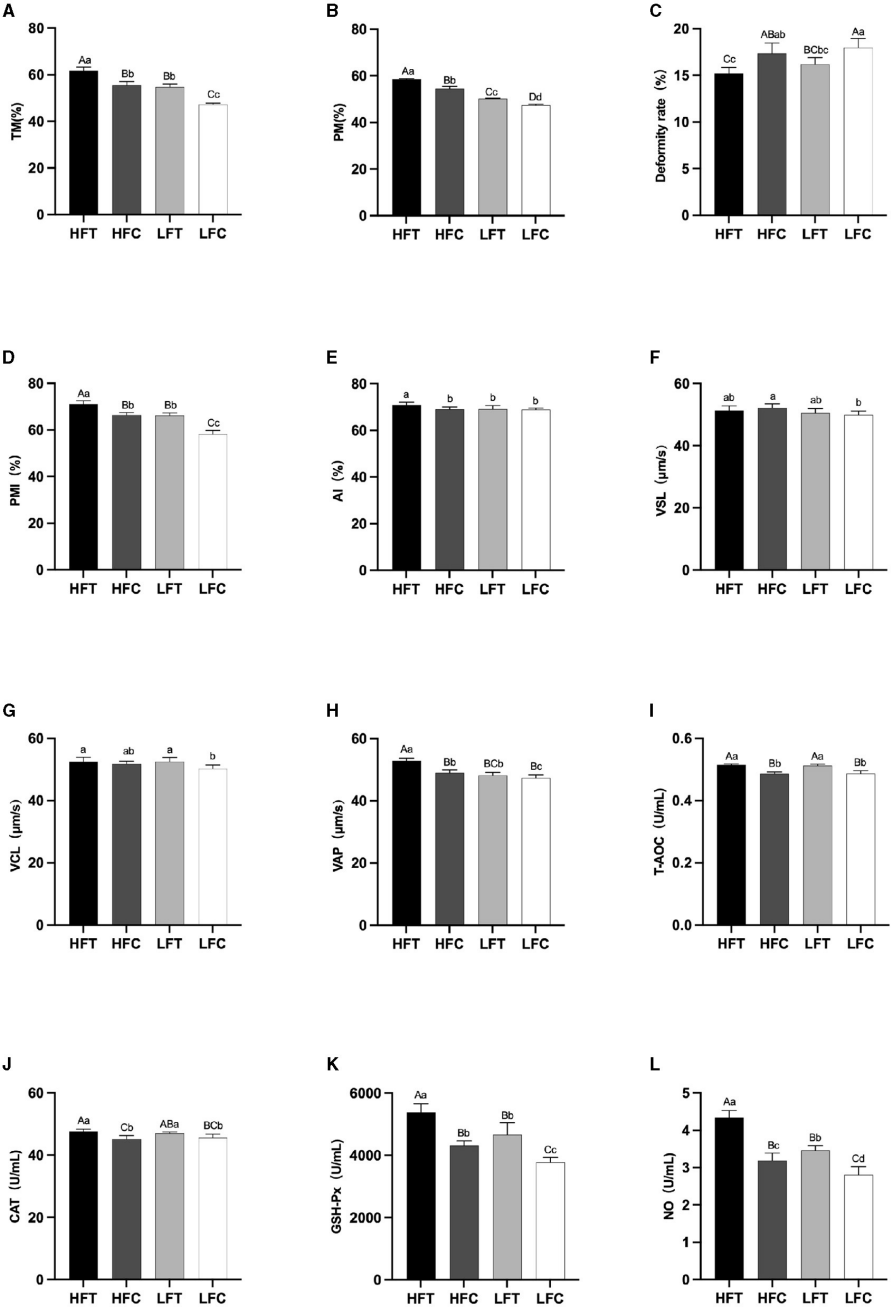
Differences in sperm parameters after thawing after supplementation with *L*-Cit. **(A)** TM, Total motility; **(B)** PM, Progressive motility; **(C)** Deformity rate; **(D)** PMI, Plasma membrane integrity, **(E)** AI, Acrosome integrity; **(F)** VSL, Velocity of straight line; **(G)** VCL, Velocity of cured line; **(H)** VAP, Velocity average path; **(I)** T-AOC; **(J)** CAT; **(K)** GSH-Px; **(L)** NO. Compared with the LFC group. Values with different lowercase superscripts mean significant difference (*P* < 0.05), while with same lowercase or no letter superscripts mean no significant difference (*P* > 0.05). Values with difference capital letter mean difference was extremely significant (*P* < 0.01).

After *L*-Cit supplementation, sperm TM (Figure 4A), PM (Figure 4B), and PMI (Figure 4D) in the LFC group exhibited significant reductions compared to the other three groups (*P* < 0.01). Furthermore, sperm Deformity rate (Figure 4C) in the LFC group was significantly elevated in comparison to both the HFT and LFT groups (*P* < 0.01). The AI (Figure 4E) and VAP (Figure 4H) in the HFT group was also significantly higher than that observed in the other three groups (*P* < 0.05). The VSL (Figure 4F) in HFC group was significantly higher than that in LFC group (*P* < 0.05). The VCL (Figure 4G) of HFT and LFT groups were significantly higher than that of LFC group (*P* < 0.05). T-AOC (Figure 4I) and CAT (Figure 4J) in both the HFT and LFT groups were significantly higher than those in the other two groups (*P* < 0.01). The GSH-Px (Figure 4K) and NO (Figure 4L) in HFT group was also significantly higher than that observed in the other three groups (*P* < 0.01). Additionally, sperm TM (Figure 4A) and PMI (Figure 4D) in the LFT group were significantly higher than those in the LFC group (*P* < 0.01).

### 3.4 Effects of *L*-Cit supplementation on the expression of HSP90 and HK1 in ram sperm

**Figure 5:**
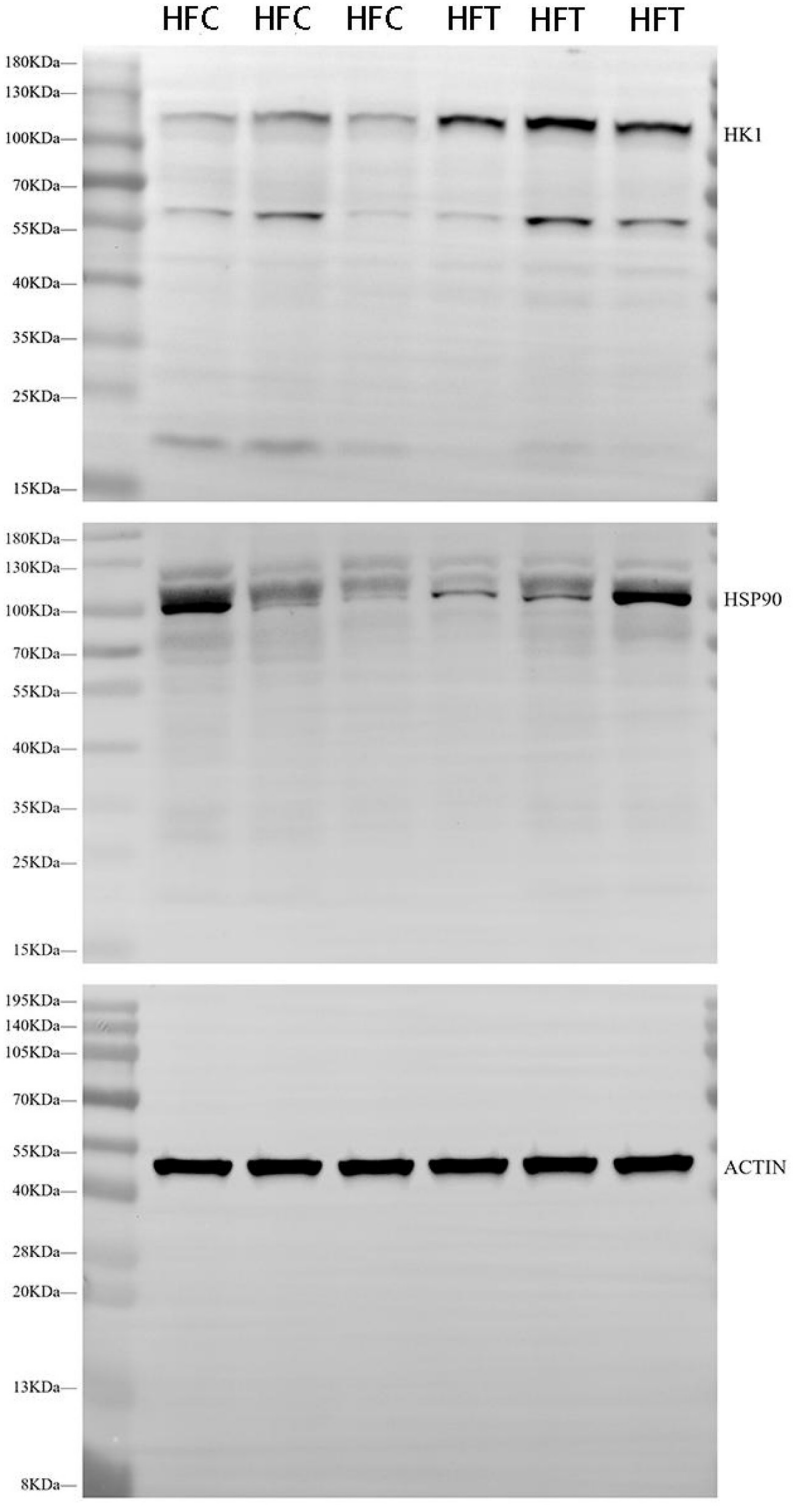
Western blotting analysis showing the protein expression of HK1 and HSP90 in frozen-thawed sheep sperm from the HFC and HFT groups.

Western blot analysis revealed significant variations in the expression levels of HK1 (Kruskal–Wallis ANOVA, df = 4, *P* = 0.016) and HSP90 (Kruskal–Wallis ANOVA, df = 4, *P* = 0.914) proteins among the groups. Refer to Figure 5 for the blots illustrating the protein expression of HK1 and HSP90 in the frozen-thawed HFC and HFT sperm.

**Figure 6:**
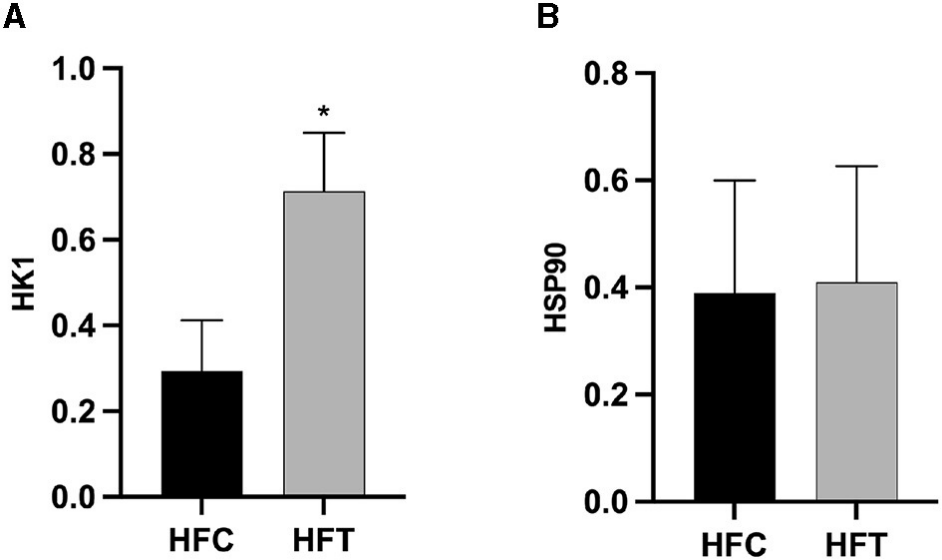
Relative expression of the **(A)** HK1 and **(B)** HSP90 proteins in sheep sperm from the HFC and HFT groups. The values represent the mean ± SEM (Standard Error of Mean). Actin was used as a control to normalize the relative expression of the analyzed proteins. Significance at *P < 0.05 after Bonferroni correction.

The bands corresponding to 115 kDa (~115 kDa) for HK1 and approximately 110 kDa (~110 kDa) for HSP90 were observed in the lysates obtained from frozen-thawed PF sperm (Figure 5). Analysis revealed variations in the expression levels of the examined proteins in the sperm of sheep in both the HFC and HFT groups (Figure 5). Compared to the HFC group, the HFT group exhibited a relatively higher (*P* = 0.016) expression of the HK1 protein after thawing (Figure 6A). There was no significant difference in HSP90 protein expression between the HFC and HFT groups (*P* = 0.914, Figure 6B).

### 3.5 Effects of *L*-Cit supplementation on spermatogenic cells in ram testis

**Figure 7:**
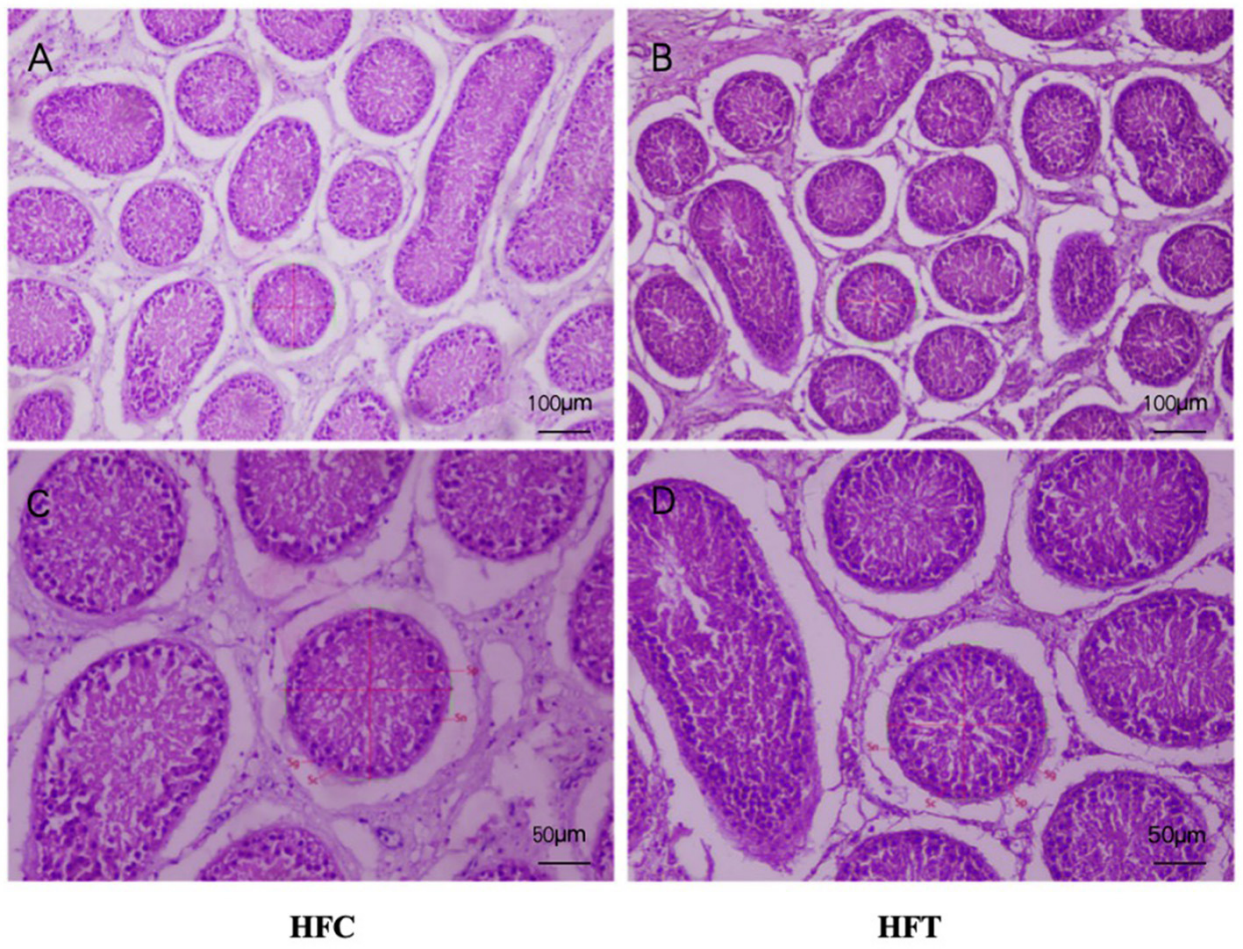
Testicular tissue sections of HFC and HFT groups. **(A, C)** were fed HFC group, **(B, D)** were HFT group. Morphological analysis after H&E staining. Sg, Spermatogonium; Sn, Sustentacular cells; Sc, Spermatocytes; Sp, Spermatids. The image above was captured at 100× using an optical microscope, and the following image was captured at 200× using an optical microscope. Significance was calculated via a *t*-test.

**Figure 8:**
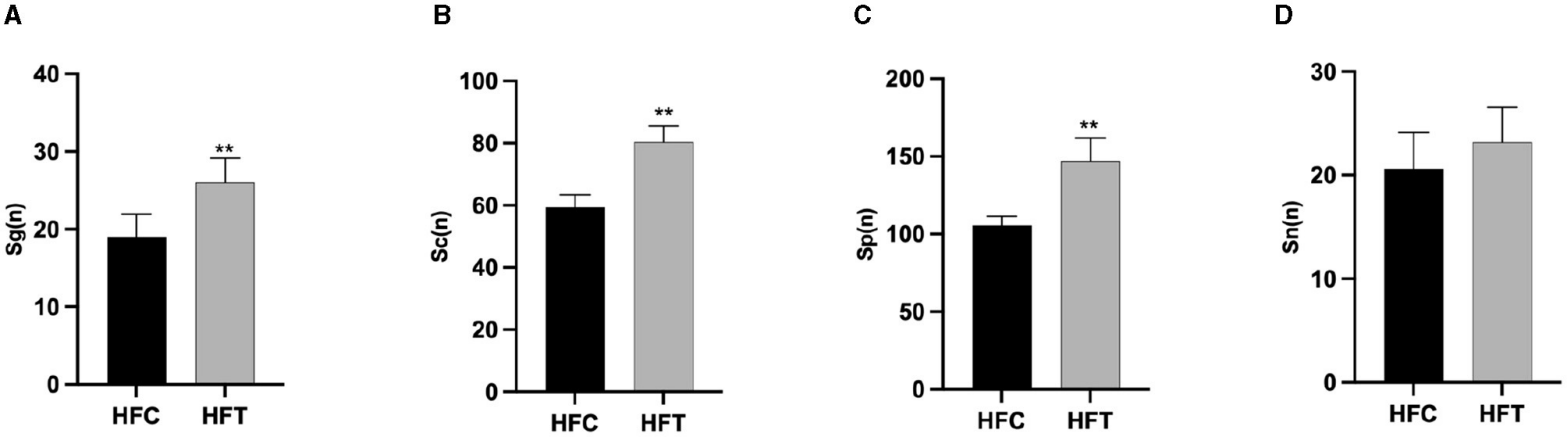
The number of Spermatogonia, Spermatocytes, Sperm cells, and Sertoli cells in HFC and HFT groups. **(A)** Sg, Spermatogonia; **(B)** Sc, Spermatocytes; **(C)** Sp, Sperm cells; **(D)** Sn, Sertoli cells. Compared with the HFC group, **P < 0.01.

The spermatogonia, Sertoli cells, spermatocytes, and sperm cells in the seminiferous tubules of ram testes from the HFT group exhibited a well-organized arrangement. Compared with the HFC group, the testicular tissue structure of the HFT group was clear and complete, the seminiferous tubules were arranged neatly, and the basement membrane was complete (Figures 7A, B). Testicular tissue samples were magnified 200-fold, as depicted in Figures 7C, D. In the HFT group, the testicular germ cells were closely arranged, and the number of germ cells and the number of germ cells in the seminiferous tubules increased (Figure 7C). Conversely, in the HFC group, testicular germ cells were loosely arranged, and the number of germ cell layers and germ cells was low (Figure 7D). Significantly higher quantities of spermatogonia (Figure 8A), spermatocytes (Figure 8B), and spermatids (Figure 8C) were observed in the HFT group compared to the HFC group (*P* < 0.05). There was no significant difference in the number of Sn (Figure 8D) between the two groups (*P* < 0.05).

### 3.6 Analysis of rumen microbial composition and diversity in rams following *L*-Cit supplementation

**Figure 9:**
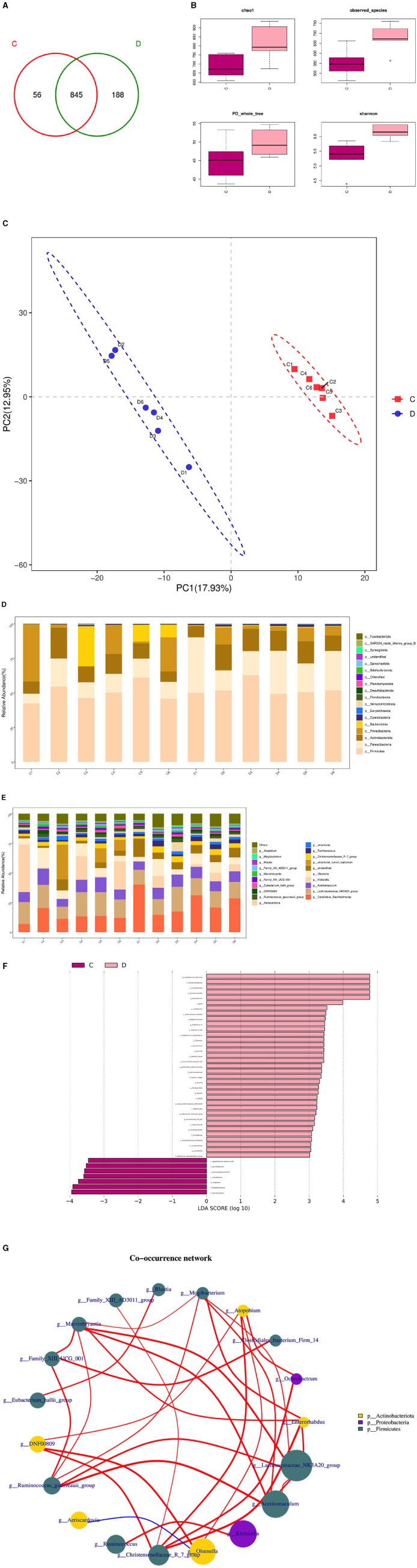
Rumen microbial composition and diversity in rams of the HFC and HFT groups. **(A)** A Wayne diagram of rumen microbial distribution comparing the two groups. **(B)** Alpha diversity index box diagram. **(C)** PLS-DA (Partial Least Squares Discrimination Analysis) analysis. **(D)** Comparison of the taxonomic composition of the rumen microbiota at the phylum level between the two groups. **(E)** Comparison of the taxonomic composition of the rumen microbiota at the genus level between the two groups. **(F)** LEfSe (LDA > 2). **(G)** Correlation analysis. C denotes the HFC group, and D indicates the HFT group.

The composition and diversity of rumen microorganisms in the HFC and HFT groups are shown in Figure 9. The number of OTUs (Operational Taxonomic Units) in the HFC and HFT groups was 56 and 188, respectively, with an overlap of 845 OTUs between both groups (Figure 9A). The average Chao1 index for rumen fluid samples in the HFC and HFT groups was 693.55 and 812.09, respectively. The average bacterial count in rumen fluid for the HFC and HFT groups was 551.28 and 680.60, respectively. The average diversity indexes for rumen fluid samples in the HFC and HFT groups were 277.57 and 303.34, respectively (Figure 9B). PCA showed distinct differences in rumen microbiota between the two groups (Figure 9C). Subsequently, we identified *Firmicutes* as the predominant microbial phylum in rumen microorganisms, followed by *Patescibacteri*a, *Actinobacteriota, Proteobacteria*, and *Bacteroidota* (Figure 9D). There was no significant difference in the abundance of *Firmicutes* at the phylum level between the two groups. The HFC group exhibited a higher abundance of *Bacteroidota*, while the HFT group showed significantly higher abundances of *Patescibacteria, Actinobacteriota*, and *Proteobacteria* (Figure 9D). The predominant genera in the rumen microflora included *Candidatus_Saccharimonas*, followed by *Lachnospiraceae_NK3A20_group, Acetitomaculum, Klebsiella*, and *Olsenella* (Figure 9E). In addition, LEFSe showed significant enrichments at the genus level, with the HFT group displaying an enrichment of *Candidatus_Saccharimonas, Staphylococcus, Weissella, Succinivbrionaceae_UcG_002*, and *Quinella*, while the HFC group exhibited an enrichment of *Ureaplasma* (Figure 9F).

The Spearman test elucidated five or more related bacterial taxa, namely *Mogibacterium, Enterorhabdus, Lachnospiraceae NK3A20, Acetitomaculum*, and *Christersenellaceae*. Furthermore, the *Family_XIII_AD3011_group* exhibited a strong association with *Blautia*, while *Mogibacterium* displayed a high association with *Christensenellaceae*. *Ochrobactrum* exhibited a strong correlation with *DNF00809*, and *Acetitomaculum* demonstrated a notable association with the *Ruminococcus_gauvreauii_group*. Conversely, the correlation between firmicutes and *Proteobacteria* was found to be relatively low. *Family_XIII_AD3011_group* displayed a significantly positive correlation with *Blautia*, while *Mogibacterium* exhibited a significant positive correlation with *Christensenellaceae*. Additionally, *Ochrobactrum* displayed a significant positive correlation with *DNF00809*, and *Acetitomaculum* exhibited a significant positive correlation with the *Ruminococcus_gauvreauii_group*. Conversely, a significant negative correlation was observed between *Firmicutes* and *Proteobacteria* (Figure 9G).

### 3.7 Analysis of microbial composition and diversity in the duodenum of rams subjected to *L*-Cit supplementary feeding

**Figure 10:**
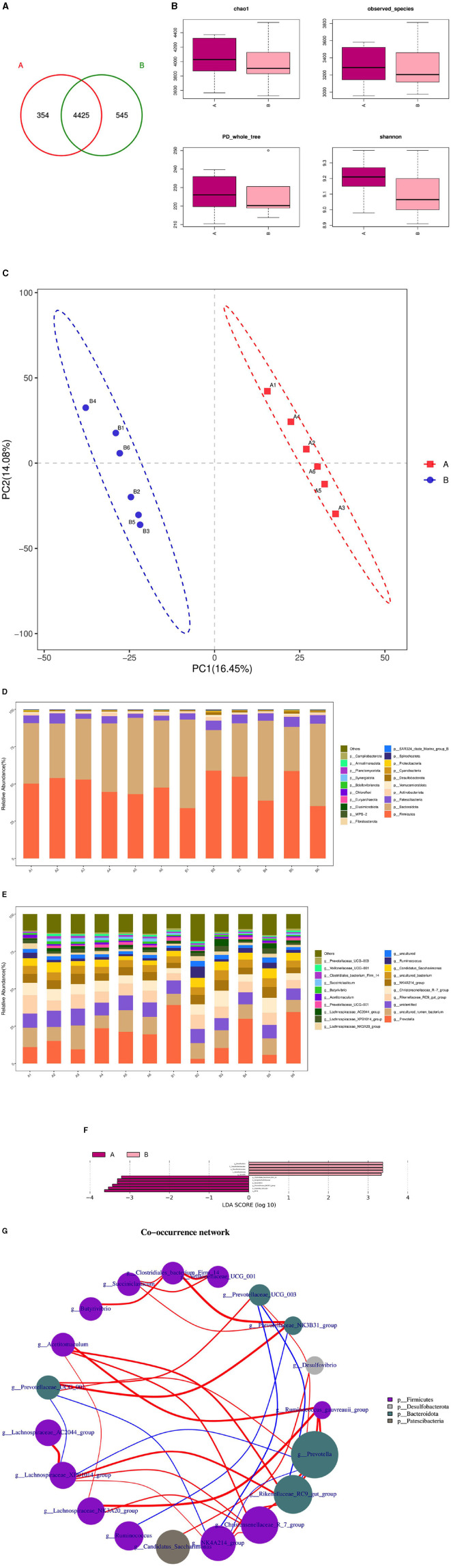
Microbial composition and diversity in the duodenum of sheep from the HFC and HFT groups. **(A)** A Wayne diagram of duodenal microbial distribution comparing the two groups. **(B)** Alpha diversity index box diagram. **(C)** PLS-DA analysis. **(D)** Comparison of the taxonomic composition of duodenal microbiota at the phylum level between the two groups. **(E)** Comparison of the taxonomic composition of duodenal microbiota at the genus level between the two groups. **(F)** LEfSe (LDA > 2). **(G)** Correlation analysis. A denotes the HFT group, and B represents the HFC group.

The microbial composition and diversity of the duodenum in the HFC and HFT groups are presented in Figure 10. The HFT group exhibited 354 OTUs, while the HFC group had 545 OTUs. Moreover, these groups shared 4,425 OTUs (Figure 10A). Regarding the average Chao1 index, the duodenal fluid samples in the HFT and HFC groups displayed values of 4,067.15 and 3,946.11, respectively. The average bacterial counts in the duodenal fluid were 3,318.17 for the HFT group and 3,278.5 for the HFC group (Figure 10B). PCA revealed a difference in duodenal flora between the two groups (Figure 10C). Subsequently, we found that the predominant microbial phylum in the duodenum was *Firmicutes*, followed by *Bacteroidota, Patescibacteria, Actinobacteriota*, and *Verrucomicrobiota* (Figure 10D). There was no significant disparity in the abundance of *Firmicutes* at the phylum level between the two groups. The HFT group exhibited higher abundances of *Bacteroidota* and *Actinobacteriota*, while the HFC group demonstrated significantly higher abundances of *Patescibacteria* and *Verrucomicrobiota* (Figure 10D). In the duodenal microflora, the predominant genus was *Prevotella*, Followed by *Uncultured rumen bacterium, Unidentified species, Rikenellaceae RC9 gut group*, and *Christensenellaceae R-7 group* (Figure 10E). In addition, LEFSe at the genus level highlighted significant enrichment of *Clostridiales bacterium Firm-14, Butyrivibrio*, and *Prevotellaceae NK3831 group* in the HFT group, while the HFC group exhibited significant enrichment of *Desulfovbrio* and *Quinella* (Figure 10F).

The Spearman test revealed that *Christersenellaceae* exhibited an association with *Lachnospiraceae_NK3A20_group, Ruminococcaceae, NK4A214_group, Acetitoma- culum*, and *Rikenellaceae_RC9_gut_group*. Conversely, *Prevotellaceae_NK3B31_ group* displayed a high correlation with *Clostridiales_bacterium_firm14*. *Prevotellaceae_UCG_003* exhibited a negative correlation with *NK4A214_group* and *Rikenellaceae_RC9_gut_group*. In addition, a negative correlation was observed between *NK4A214_group* and *Prevotella*, as well as between *Prevotellaceae_UCG_001* and *NK4A214_group*. Furthermore, a negative correlation was identified between *Prevotellaceae_UCG_001* and *Lachnospiraceae_XPB1014_group*, as well as between *Lachnospiraceae_XPB1014_group* and *Prevotella*, and finally, between *Prevotella* and *Rikenellaceae_RC9_gut_group* (Figure 10G).

### 3.8 Analysis of the association between *L*-Cit supplementation and rumen and duodenal microbiota in rams

**Figure 11:**
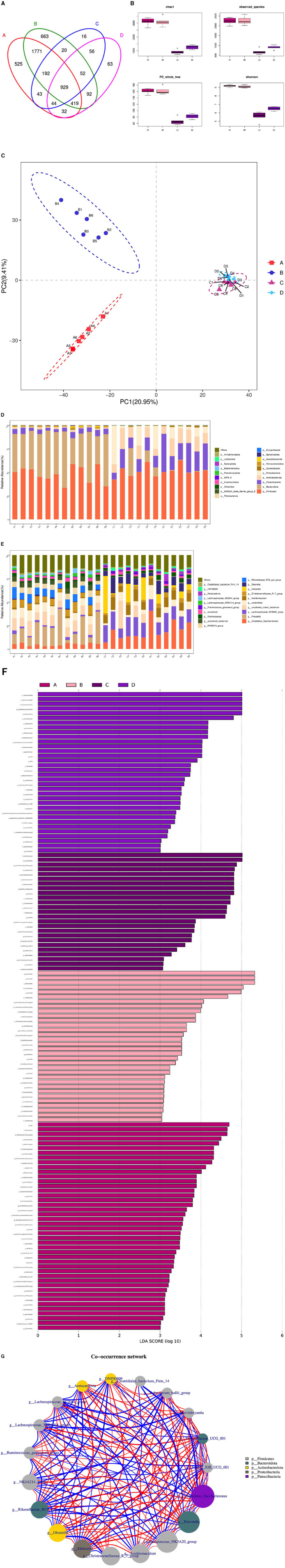
Correlation analysis of rumen and duodenal microbiota in the HFC and HFT groups. **(A)** A Wayne diagram of microbial distribution comparing the two groups. **(B)** Alpha diversity index box diagram. **(C)** PLS-DA analysis. **(D)** Comparison of the taxonomic composition of the microbiota at the phylum level between the two groups. **(E)** Comparison of the taxonomic composition of the microbiota at the genus level between the two groups. **(F)** LEfSe (LDA > 2). **(G)** Correlation analysis. A and B indicate duodenal microbiota in the HFT and HFC groups, respectively, and C and D indicate rumen microbiota in the HFC and HFT groups, respectively.

The microbial associations in the rumen and duodenum of the HFC and HFT groups are shown in Figure 11. The OTUs identified in the rumen and duodenum of the HFC and HFT groups were 16, 63, 663, and 525, respectively, resulting in a total of 929 OTUs (Figure 11A). The average Chao1 index values for the rumen and duodenum samples in the HFC and HFT groups were 920.48, 1,268.25, 3,096.13, and 3,110.84, respectively. Furthermore, the average bacterial counts in the rumen and duodenal fluid of the HFC and HFT groups were 683.27, 899.58, 2,253.07, and 2,237.72 (Figure 11B), respectively. PCA demonstrated differences in the flora of the rumen and duodenum between the two groups (Figure 11C). Subsequently, we observed that *Firmicutes*, followed by *Bacteroidota, Patescibacteria, Actinobacteriota*, and *Proteobacteria* (Figure 11D), were the predominant phyla in both the rumen and duodenum. At the phylum level, no significant differences were detected in the abundance of *Firmicutes* between the rumen and duodenum of the two groups. However, the duodenum exhibited a higher abundance of *Bacteroidota*. Additionally, the rumen of the HFC group displayed significantly higher levels of *Proteobacteria*, while the rumen of the HFT group exhibited a significantly higher abundance of *Patescibacteria* and *Actinobacteriota* (Figure 11D). The predominant genera in the microbial flora included *Candidatus_Saccharimonas, Prevotella, Lachnospiraceae_ NK3A20_group, Uncultured_rumen_bacterium*, and *Unidentified species* (Figure 11E). In addition, LEFSe highlighted significant enrichments at the genus level, with the HFC group showing significant enrichment in *Lachnospiraceae_NK3A20_group, Acobitomaculum, Klebsiella*, and *Olsenella*, and the HFT group displaying significant enrichment in *Candidatus_Saccharimonas, Family_XIII_AD3011_group, Ochrobactrum*, and *Atopobium*. In the duodenum, the HFC group exhibited significant enrichments in *Lachnospiraceae_AC2044_group, Prevotellaceae_XPB1014_group, Prevotellaceae_UGG_003*, and *Desulfovibrio*, while the HFT group displayed significant enrichments in *Rikenellacase_PC9_gut_group, NK4A214_group, Christensenellaceae_R-7_group*, and *Succinictasticum* (Figure 11F).

The Spearman test revealed that *Prevotellaceae_UGG_001* and *Marvinbryantia, Eubacterium_hallii_group, Family_III_UCG_001, Candidatus_Saccharimonas, Rikenellaceae_RC9_gut_group, Lachnospiraceae_AC2044_group, Family_III_UCG_ 001, Candidatus_Saccharimonas, Klebsiella, Prevotella, Lachnospiraceae_AC2044_ group*, and *Prevotellaceae_XPB101_group* were highly associated with *Ruminococcus_gauvreauii_group* and *DNF00809*. *Candidatus_Saccharimonas* was negatively correlated with *Clostridiales_bacterium_Firm_14, Lachnospiraceae_AC2044_group*, and *Lachnospiraceae_ XPB1014_group*. In addition, *Lachnospir-aceae_NK3A20_group, Clostridiales_bacterium_Firm_14, Christensenellaceae_R_7_ group, Olsenella, Rikenellaceae_RC9_gut_group, Eubacterium_hallii_group, DNF00809, Aeriscardovia, Ruminococcus_gauvreauii_group, NK4A214, Eubacterium_hallii_group*, and R*uninococcus_gauvreauii_group* were negatively correlated with *Lachnospiraceae_XPB1014_group* and *Lachnospiraceae_AC2044_group* (Figure 11G).

### 3.9 Correlation analysis between rumen flora and semen quality of rams after supplementary feeding with *L*-Cit

**Figure 12:**
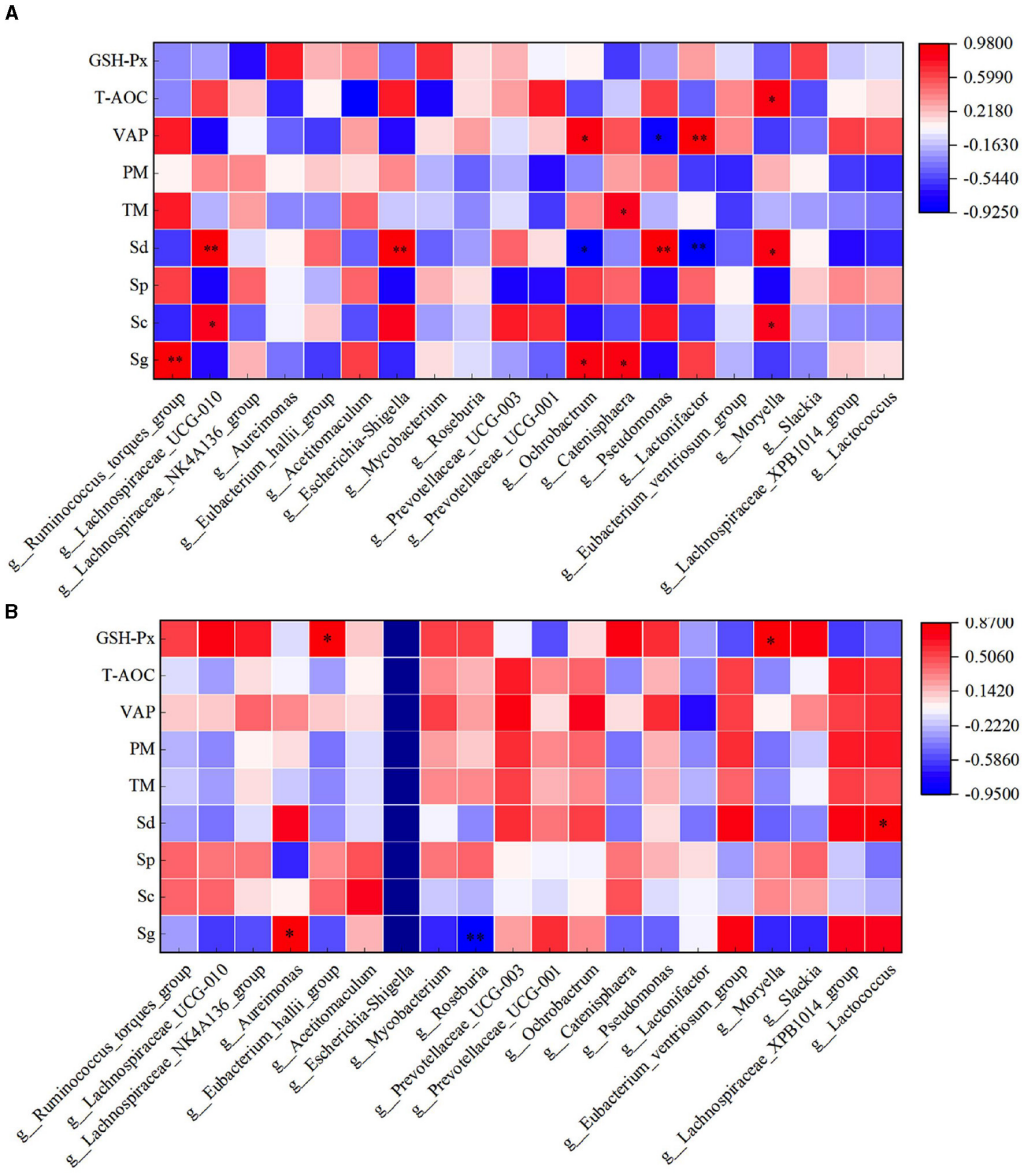
Correlation analysis of spermatogenic cells, fresh sperm index, and rumen flora in the HFC and HFT groups. **(A)** indicates the HFC group, and **(B)** denotes the HFT group. ^*^indicates a significant difference between groups where *P* < 0.05. ^**^indicates a significant difference between groups where *P* < 0.01.

Through correlation analysis of spermatogenic cell count, fresh sperm index, and rumen flora, the number of Sg exhibited a significant positive correlation with the abundance of *Ruminococcus_torques_group, Ochrobactrum*, and *Catenisphaera* in the HFC group (*P* < 0.05). Furthermore, the number of Sc showed a significant positive correlation with the abundance of *Lachnospiraceae_UCG-010* and *Moryella* (*P* < 0.05). The abundance of Sd demonstrated a significant positive correlation with the abundance of *Lachnospiraceae_UCG-010, Escherichia_Shigella*, and *Pseudomonas* (*P* < 0.01), while exhibiting a significant negative correlation with the abundance of *Ochrobactrum* and *Lactonifactor* (*P* < 0.05). TM was significantly positively correlated with the abundance of *Catenisphaera* (*P* < 0.05), while the VAP was positively correlated with the abundance of *Ochrobactrum* and *Lactonifactor* (*P* < 0.05). Additionally, T-AOC exhibited a positive correlation with *Moryella* abundance (*P* < 0.05). In the HFC group, the number of Sg displayed significant positive correlations with the abundance of *Aureimonas* (*P* < 0.05) and *Roseburia* (*P* < 0.01). The number of Sd also exhibited a significant positive correlation with the abundance of *Lactococcus* (*P* < 0.05), and the concentration of GSH-Px was significantly positively correlated with the abundance of *Eubacterium_hallii_group* and *Moryella* (*P* < 0.05) (Figure 12).

**Figure 13:**
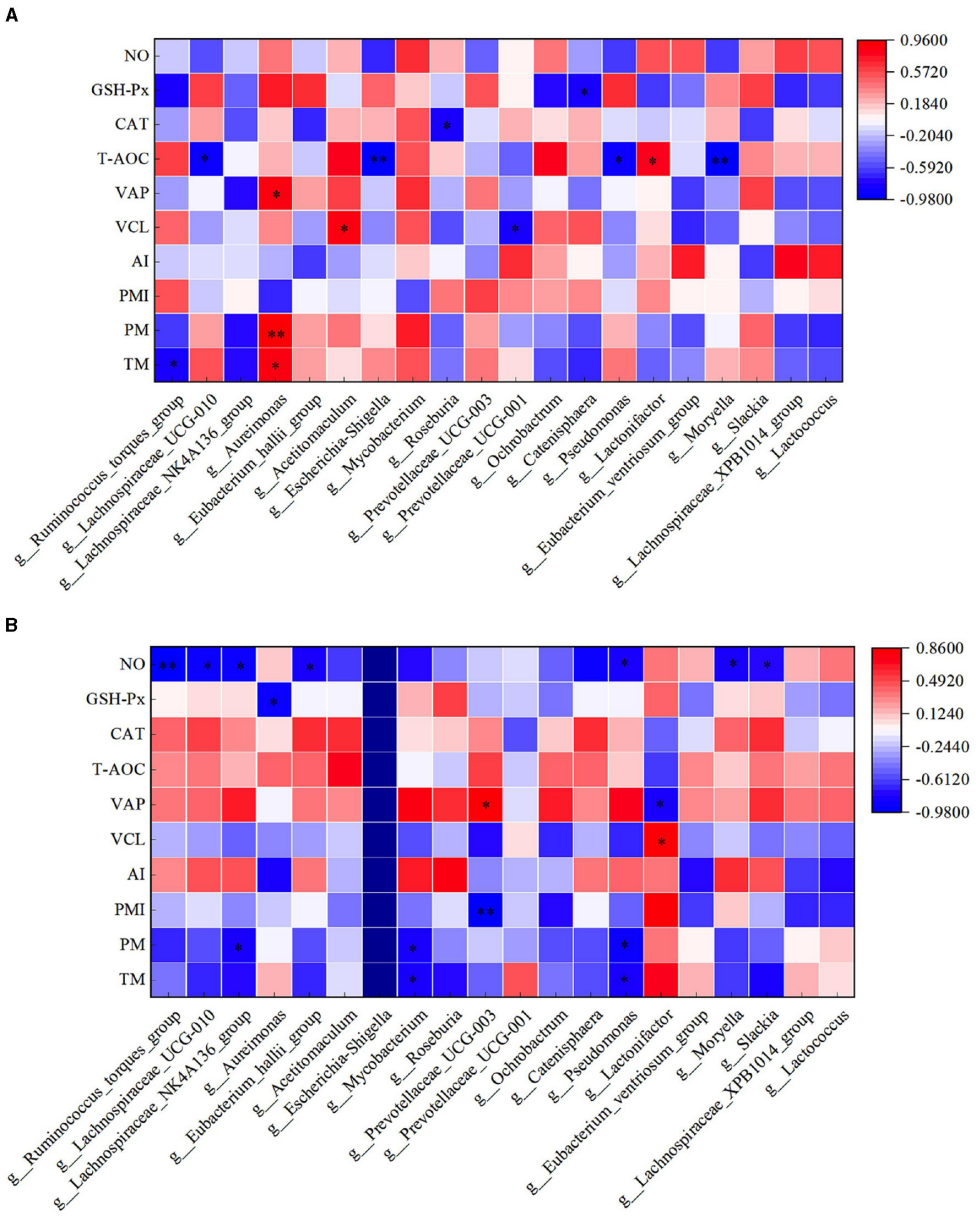
Correlation analysis between semen parameters and rumen flora after thawing in HFC and HFT groups. **(A)** is HFC group, **(B)** is HFT group. ^*^indicates a significant difference between groups where *P* < 0.05. ^**^indicates a significant difference between groups where *P* < 0.01.

In the HFC group, both TM and PM exhibited significant positive correlations with the abundance of *Aureimonas* (*P* < 0.05). VCL displayed a significant positive correlation with the abundance of *Acetitomaculum* (*P* < 0.05) and a significant negative correlation with the abundance of *Prevotellaceae_UCG-001* (*P* < 0.05). Furthermore, VAP demonstrated a significant positive correlation with the abundance of *Aureimonas* (*P* < 0.05). T-AOC content was significantly negatively correlated with the abundance of *Lachnospiraceae_UCG-010, Escherichia_Shigella, Pseudomonas*, and *Moryella* (*P* < 0.05). CAT content displayed a significant negative correlation with *Roseburia* abundance (*P* < 0.05), and GSH-Px concentration was significantly negatively correlated with *Catenisphaera* abundance (*P* < 0.05). In the HFT group, both TM and PM were significantly negatively correlated with the abundance of *Mycobacterium* and *Pseudomonas* (*P* < 0.05). PMI showed a significant negative correlation with the abundance of *Prevotellaceae_UCG-003* (*P* < 0.01). VCL exhibited a significant positive correlation with the abundance of *Lactonifactor* (*P* < 0.05), and VAP displayed a significant positive correlation with the abundance of *Prevotellaceae_UCG-003* (*P* < 0.05). Moreover, NO concentration was significantly negatively correlated with the abundance of *Ruminococcus_torques_group, Lachnospiraceae_UCG-010, Eubacterium_hallii_group, Pseudomonas*, and *Moryella* (*P* < 0.05) (Figure 13).

### 3.10 Correlation analysis between duodenal flora and semen quality of rams after supplementary feeding with *L*-Cit

**Figure 14:**
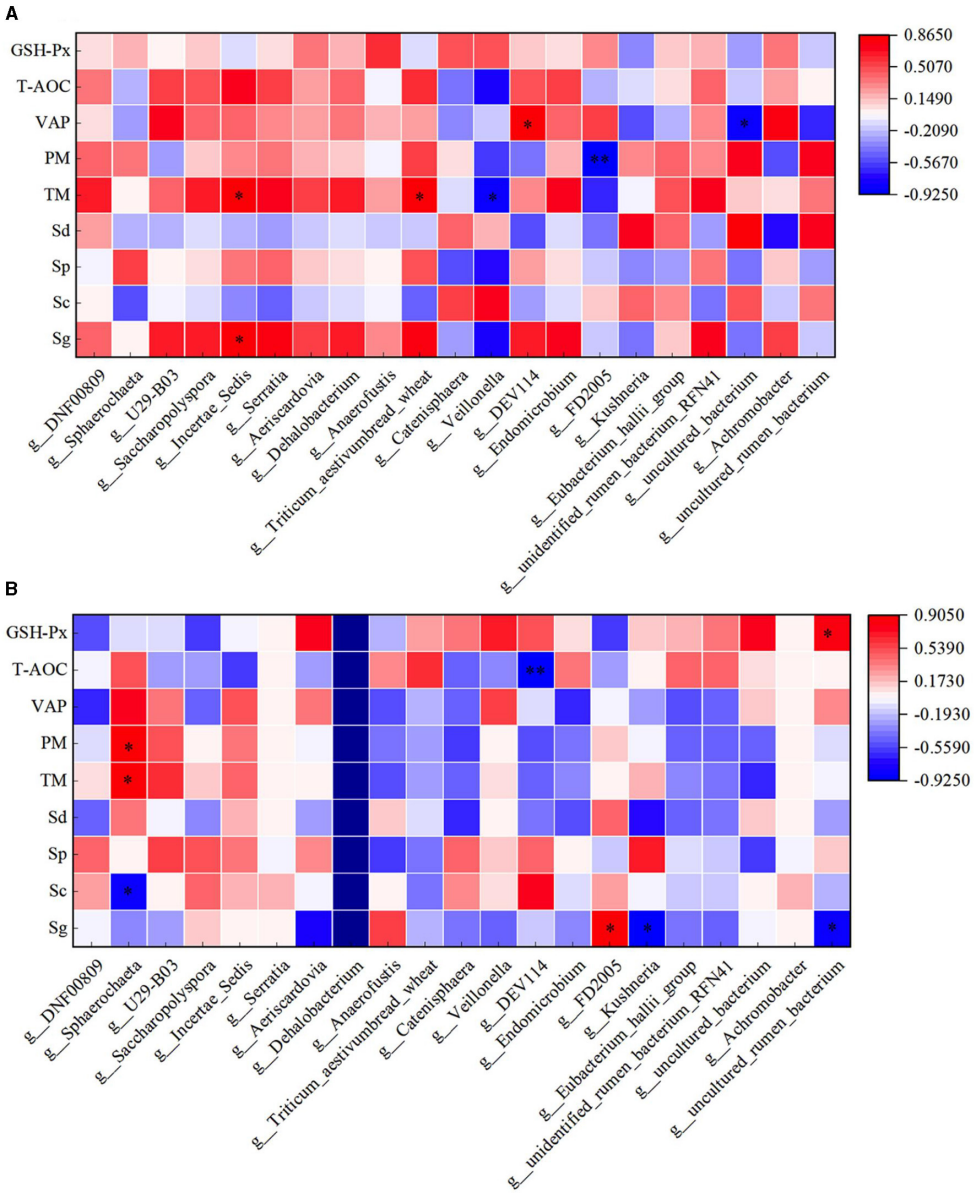
Correlation analysis of spermatogenic cells, fresh sperm index, and duodenal flora in the HFC and HFT groups. **(A)** denotes the HFC group, and **(B)** represents the HFT group. ^*^indicates a significant difference between groups where *P* < 0.05. ^**^indicates a significant difference between groups where *P* < 0.01.

Through correlation analysis of spermatogenic cell count, fresh sperm index and duodenal flora, the number of Sg displayed exhibited a positive correlation with the abundance of *Incertae_Sedis* (*P* < 0.05), and TM exhibited a positive correlation with the abundance of *Incertae_Sedis* and *Triticum aestivumbread_wheat* (*P* < 0.05). There exhibiting a significant negative correlation with *Veillonella* abundance (*P* < 0.05), VAP exhibited a positive correlation with the abundance of *DEV114* (*P* < 0.05), and exhibiting a significant negative correlation with the abundance of *Unclutured_bacterium*. PM exhibiting a significant negative correlation with *FD2005* abundance (*P* < 0.01). In the HFT group, the abundance of *Sphearochaeta* exhibiting a significant negative correlation with the number of Sc (*P* < 0.05), and exhibited a positive correlation with TM and PM (*P* < 0.05). The abundance of *DEV114* exhibiting a significant negative correlation with T-AOC content (*P* < 0.01). The abundance of Sg exhibited a positive correlation with the abundance of *FD2005* (*P* < 0.05), and exhibiting a significant negative correlation with the abundance of *Kushneria* (*P* < 0.05). The abundance of *Uncultured_rumen_bacterium* exhibiting a significant negative correlation with the number of Sg (*P* < 0.05), and exhibited a positive correlation with the content of GSH-Px (*P* < 0.05) (Figure 14).

**Figure 15:**
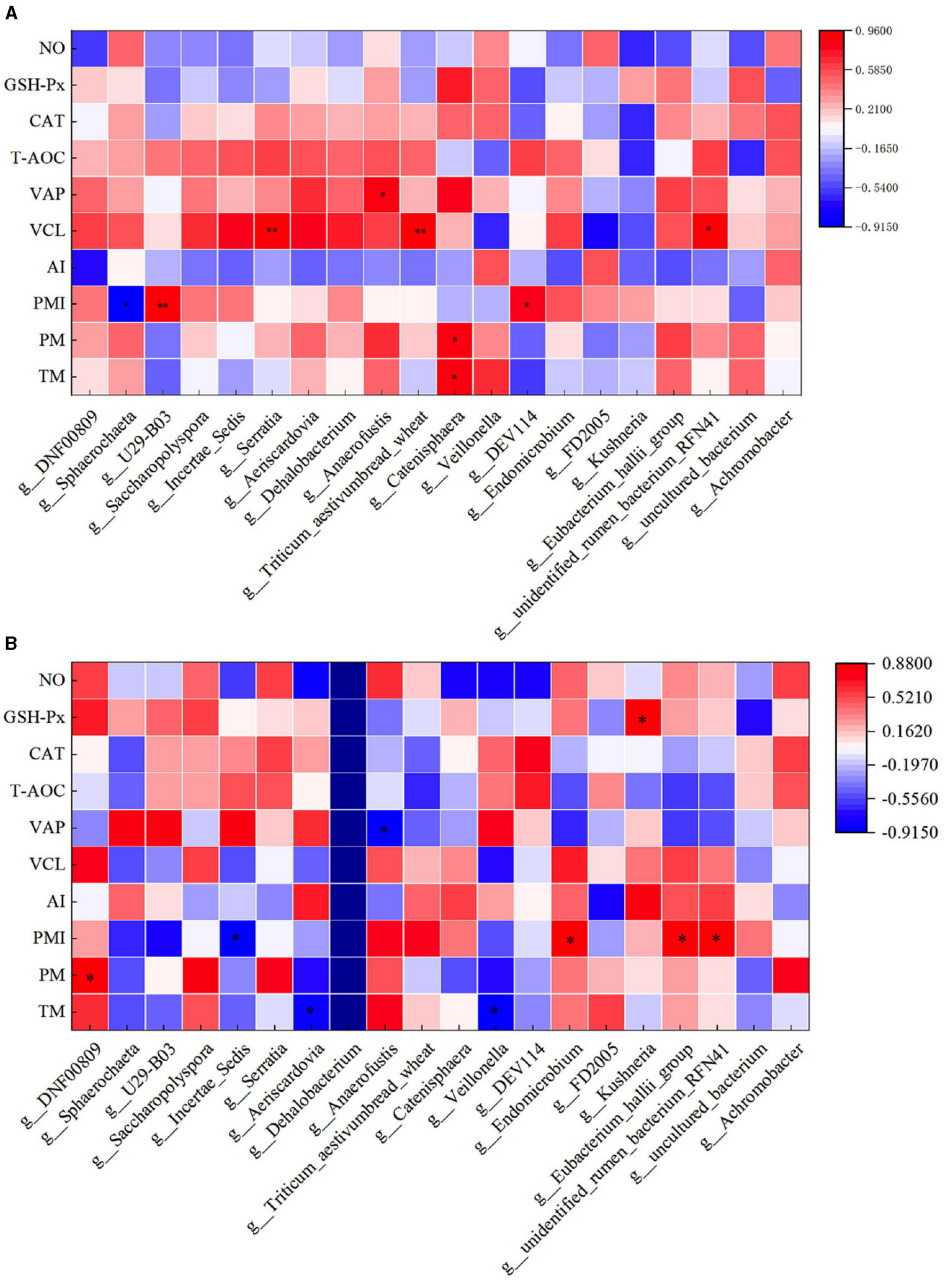
Correlation analysis between semen parameters and duodenal flora after thawing in the HFC and HFT groups. **(A)** denotes the HFC group, and **(B)** represents the HFT group.

The correlation analysis between semen parameters and duodenal flora following freezing and thawing indicated that in the HFC group, TM and PM exhibited significant positive correlations with the abundance of *Catenisphaera* (*P* < 0.05). Additionally, PMI was significantly positively correlated with the abundance of *U29-B03* and *DEV114* (*P* < 0.05) but significantly negatively correlated with the abundance of *Sphaerochaeta* (*P* < 0.05). VCL was significantly positively correlated with the abundance of *Serratia, Triticum_aestivumbread_wheat*, and *Unidentified_rumen_bacterium_RFN41* (*P* < 0.05), while VAP demonstrated a positive correlation with *Anaerofustis* abundance (*P* < 0.05). In the HFT group, TM exhibited a significant negative correlation with the abundance of *Aeriscardovia* and *Veillonella* (*P* < 0.05). Moreover, a significant positive correlation was observed between PM and *DNF00809* abundance (*P* < 0.05). PMI showed a negative correlation with the abundance of *Incertae_Sedis* (*P* < 0.05) and a positive correlation with the abundance of *Endomicrobium, Eubacterium_hallii_group*, and *Unidentified_rumen_bacterium_RFN41* (*P* < 0.05). Furthermore, VAP displayed a negative correlation with *Anaerofustis* abundance (*P* < 0.05). The levels of GSH-Px exhibited a positive correlation with the abundance of *Kushneria* (*P* < 0.05) (Figure 15).

## 4 Discussion

Cryopreservation of semen holds significant importance in safeguarding sheep germplasm resources and advancing artificial insemination technology. Upon isolation, semen comes into contact with the outside air, it leads to excessive production of reactive oxygen species (ROS), which has a harmful effect on sperm cells. In addition, low temperatures can cause irreversible damage to the sperm's plasma membrane, acrosome, and DNA integrity, ultimately diminishing the quality of frozen semen and greatly reducing sperm fertilization potential (Yánez-Ortiz et al., 2022). Under normal circumstances, seminal plasma contains antioxidants such as CAT, SOD (Superoxide Dismutase), and GSH-Px, which mitigate ROS levels, thereby reducing sperm damage (Gadea et al., 2010). However, semen freezing induces an overproduction of ROS, and the low level of endogenous antioxidants in seminal plasma leads to oxidative stress-induced damage to sperm cells. Therefore, exogenous antioxidants are frequently added to the diluent to alleviate oxidative damage to sperm cells (Zhang et al., 2012). In this study, *L*-Cit feeding led to an enhancement in the antioxidant capacity of ram semen, correlating with increased sperm motility after freezing. This suggests that *L*-Cit can boost the antioxidant capacity of ram semen and subsequently enhance the sperm's resistance to freezing. Cryopreservation of sperm can induce alterations in sperm cell membranes and their components, lead to decreased sperm motility and fertilization rate potential upon thawing (Upadhyay et al., 2021). However, different individuals' semen exhibit varying abilities to withstand freezing-induced damage (Rickard et al., 2016). Our previous study demonstrated that *L*-Cit supplementation results in elevated levels of most amino acids in the seminal plasma of rams, alongside reduced carbohydrate levels. These changes may exert a buffer effect against physical damage during sperm freezing, thereby improving the antifreeze performance of sperm. However, this mechanism requires further examination (Zhao et al., 2023).

*L*-Cit can be synthesized in the intestinal tract of adult animals; however, it does not participate in protein synthesis *in vivo* (Gilbreath et al., 2019). *L*-Cit is not degraded in the rumen of sheep and cattle and can subsequently be transported to the kidney as a precursor to arginine, thus fulfilling the body's arginine requirements (Gilbreath et al., 2020). Functioning as an intermediate product in the urea cycle, *L*-Cit not only improves the body's antioxidant capacity but also elevates arginine and NO levels (Fragkos and Forbes, 2018). NO is essential for the functioning of the testis, epididymis, and vas deferens, facilitating penile erection and vasodilation (Wu et al., 2017). Serving as a precursor to both arginine and NO, *L*-Cit augments the levels of arginine and NO upon entry into the body. In this study, *L*-Cit improved semen collection and exhibited antioxidant properties in rams. These effects may be attributed to *L*-Cit's metabolic synthesis of arginine and NO (Miraglia et al., 2011). Stanislavov and Rohdewald (2014) showed that *L*-Cit, along with arginine, can improve sperm motility and density. The current research results show that, prior to the supplementation of *L*-Cit, the curvilinear motion rate of sperm in the HF group was significantly higher than that in the LF group, with no significant disparity in the linear motion rate. This may be due to the HF group exhibited higher sperm motility, it also had a greater number of sperm displaying curvilinear motion, resulting in a significantly higher curvilinear motion rate of sperm in the HF group compared to the LF group. After freeze-thawing, we observed significantly higher levels of TM, PM, VAP, GSH-Px, and NO activities in the sperm of the HF group compared to those in the LF group. This may be due to the elevated antioxidant content in the HF group, thereby enabling semen in the HF group to mitigate the adverse effects of low temperatures on sperm and improve semen quality. Following *L*-Cit supplementary feeding, we observed significantly increased sperm density, TM, T-AOC, GSH-Px, and NO activity in fresh semen in the experimental group as compared to those in the control group, both in the HF and LF groups. After freeze-thawing, we found significantly higher levels of TM, PM, PMI, T-AOC, CAT, GSH-Px, and NO activity in sperm in the experimental group than those in the control group. This may be attributed to the *L*-Cit supplementary feeding, which raised arginine and NO levels in rams. *L*-Cit may be involved in sperm production via the arginine and NO metabolic pathways in the body (Cherney et al., 2003). Therefore, we speculate that *L*-Cit supplementation has the potential to enhance semen antioxidant capacity, subsequently reducing the deleterious effects of ROS on sperm and thereby improving semen quality following freezing and thawing.

Studies have found that *L*-Cit can effectively improve the body's nitrogen balance. *L*-Cit enhances muscle protein synthesis and muscle deposition, attenuates bodily damage, and augments the body's antioxidant capacity (Liu et al., 2020). Simultaneously, supplementary feeding of *L*-Cit can significantly improve the levels of *L*-valine and *L*-arginine in ram seminal plasma, substantiating its potential to promote amino acid synthesis and metabolism in the body (Zhao et al., 2022). During cryopreservation, sperm motility, effective survival time, sperm concentration, and antioxidant enzyme activity reduce by approximately 40%−50% (Hu et al., 2011). Studies have shown that the freeze-thawing process can compromise the functionality of sperm proteins (Martin et al., 2007) and that certain proteins in sperm are pivotal for sustaining sperm motility post-freezing and thawing (Tsou, 2000). HK1, acting as a transferase, utilizes ATP to convert glucose into glucose-6-phosphate during glycolysis (Nakamura et al., 2008a). *In vitro*, glycolysis represents the primary source of ATP in mammalian sperm, supplying the majority of ATP required for sperm motility. Several glycolytic regulatory enzymes have been found to be located in the middle of the sperm flagella, including HK1, glucose phosphate isomerase, phosphofructokinase 1, aldolase, and enolase. These enzymes play a crucial role in maintaining sperm functionality (Vemuganti et al., 2007). Studies have elucidated alterations in the content of glycolytic regulatory enzymes in sperm with impaired motility (Force et al., 2002). Western blot analysis has revealed the abundance of HK1 in mouse sperm, with immunohistochemistry validating its predominant localization in the midsection of sperm (Vemuganti et al., 2007). The hexokinase activity of mouse sperm isolated from the epididymis was lower than that of sperm after a 5-min incubation, indicating a direct correlation between HK1 and sperm motility (Nakamura et al., 2008b). In this study, TM, PM, and VAP in the HFT group were significantly higher than those in the HFC group. In addition, the HK1 content in the HFT group was also significantly higher than that in the HFC group, which may be due to *L*-Cit promoting the synthesis of HK1 in the body, increasing the HK1 content of semen, elevating the ATP levels in sperm, and improving sperm motility.

ATP is generated through mitochondrial oxidative phosphorylation and glycolysis, providing energy for sperm motility (Ashrafzadeh et al., 2013). Studies have identified dihydrolipoamide dehydrogenase precursor, fumaric acid hydratase precursor, and thiotransferase as catalysts in the energy production pathway, promoting ATP production and enhancing sperm motility (Martínez-Heredia et al., 2008). Recent studies have also illuminated the involvement of HSP90 in ATP metabolism (Prodromou et al., 1997). The reduction of HSP90 during the freezing process may lead to decreased sperm motility due to ATP depletion. In addition, HSP90 has the capacity to activate nitric oxide synthase and improve sperm motility (García-Cardeña et al., 1998). The expression of HSP90 is proportional to sperm motility, PMI, and AI (Zhang et al., 2015). In this study, the sperm PMI, AI, and VAP in the HFT group were significantly higher than those in the LFC group after thawing. However, we observed no significant difference in HSP90 expression between the two groups. This lack of distinction may be attributed to the broad participation of the persons involved. HSP90 in ATP metabolism during freezing in HFT group may lead to the depletion of HSP90 content. Alternatively, it could be due to the fact that *L*-Cit is not involved in the synthesis of HSP90, resulting in comparable HSP90 content between the semen of the HFT and HFC groups.

The primary structural component of the testis is the seminiferous tubule, and each seminiferous tubule is coiled around it, collectively constituting the internal structure of the testis. The histological morphology of seminiferous tubules effectively and intuitively reflects the testis's developmental stage and reproductive capability (Hermann et al., 2018). Wei et al. found that supplementing arginine in the diet significantly increased the testicular volume and weight in boars. Furthermore, arginine supplementation resulted in denser germ cell arrangement, a significant increase in spermatogenic epithelium thickness, and a significant rise in spermatogonia count within the seminiferous tubules (Wei et al., 2022). De et al. (2016) found that arginase inhibitors raised *L*-arginine levels by inhibiting the arginase pathway, thereby increasing germ cell numbers. Additionally, supplementing *L*-Cit, an arginine precursor, elevated *L*-arginine content in ram seminal plasma (Gadea et al., 2010). In this study, the testicular germ cells in the HFT group were closely arranged, and the number of germ cells and the number of germ cells in the seminiferous tubules were more than those in the HFC group, validating that *L*-Cit supplementation can stimulate seminiferous tubule and germ cell development. Spermatogonia are primordial germ cells located in the seminiferous epithelium, and their deformation eventually transforms them into sperm formation (Hess and Renato de Franca, 2008). That is, the increase in the number of spermatogonia will lead to an increase in the number of sperm. Chen et al. demonstrated that adding 0.8%−11.0% *L*-arginine to boar diets significantly improved sperm count and semen antioxidant capacity (Chen et al., 2018). This suggests that arginine can significantly improve the reproductive performance of male animals, possibly by promoting spermatogonia proliferation. In this study, the sperm density, viability, motility, and antioxidant capacity of semen in the HFT group were significantly higher than those in the HTC group. This may be due to the fact that *L*-Cit supplementation increased *L*-arginine content in the rams, promoted the proliferation of spermatogonia, and enhanced the antioxidant capacity of semen, thereby improving the quality of semen and improving the cryopreservation effect of semen in rams to a certain extent.

The gut microbiota is involved in various aspects of host health. The gut microbiota can regulate semen quality through its influence on host amino acid metabolism and energy metabolism (Wang et al., 2023). Studies have shown that augmenting the concentration of dietary concentrate can upregulate the expression of VFA absorption-related genes in the rumen epithelium of goats, thereby enhancing the rumen epithelium's capacity to absorb VFAs (Schürmann et al., 2013). This phenomenon may be linked to the increase in VFA concentration and the reduction in pH in the rumen fluid. Increasing the proportion of dietary concentrate results in a higher VFA concentration, thereby improving the production performance of ruminants (Prieto and Verdugo, 2018). However, VFAs, being weak acids, primarily undergo electrolysis in the rumen, releasing H^+^, and consequently lowering the pH of the rumen fluid. When the VFA concentration surpasses the rumen's capacity to neutralize the released H^+^ it can lead to rumen acidosis (Kleen and Cannizzo, 2012). In this study, the enhancement of semen quality by *L*-Cit supplementation can be attributed to the improvement of intestinal flora abundance by *L*-Cit supplementation and the stimulation of gene expression associated with VFA absorption in the rumen epithelium. This, in turn, improves the absorption and utilization of VFAs, ultimately contributing to improved semen quality. Studies have shown that dietary supplementation with arginine can promote the synthesis of Ca^2+^ and cAMP (Cyclic adenosine monophosphate), thereby improving semen quality in boars (Dong et al., 2016). Amino acids play a crucial role in regulating intestinal flora. It has been reported that leucine can promote intestinal development in piglets (Zhou et al., 2018). Glutamate can significantly influence the composition of the intestinal microbial community, enhance microbial diversity, and facilitate the colonization of *Roseburia* (Feng et al., 2015).

After supplementation with *L*-Cit in this experiment, the OTUs in the rumen of HFC and HFT groups were 56 and 188, respectively, and the OTUs in the duodenum were 545 and 354, respectively, indicating that supplementation with *L*-Cit can enrich the abundance of bacteria in the rumen of rams and reduce the abundance of duodenal flora. The reason may be that *L*-Cit increases the microorganisms in the rumen that are easily digested and metabolized by the duodenum and increases the body's bacterial protein. Through the correlation analysis of the number of spermatogenic cells, fresh sperm index and gastrointestinal flora, it was found that the flora affecting spermatogenic cells and semen quality was inconsistent between the two groups. This may be due to the fact that *L*-Cit promoted the abundance of beneficial bacteria in the rumen, promoted the production of VFA or increased the body's bacterial protein, thereby improving semen quality. Ding et al. (2020) found that there was a significant negative correlation between the abundance of *Bacteroides* and *Prevotella* and sperm motility. In addition, the abundance of *Bacteroides* showed a strong positive correlation with the concentration of endotoxin in the patient's blood. In this study, the abundance of *Bacteroidota* was higher in the HFC group after supplemental feeding of *L*-Cit, indicating that *L*-Cit could reduce the abundance of bacteroidetes in the rumen, which may improve sperm quality by reducing the level of endotoxin in the blood.

**Figure 16:**
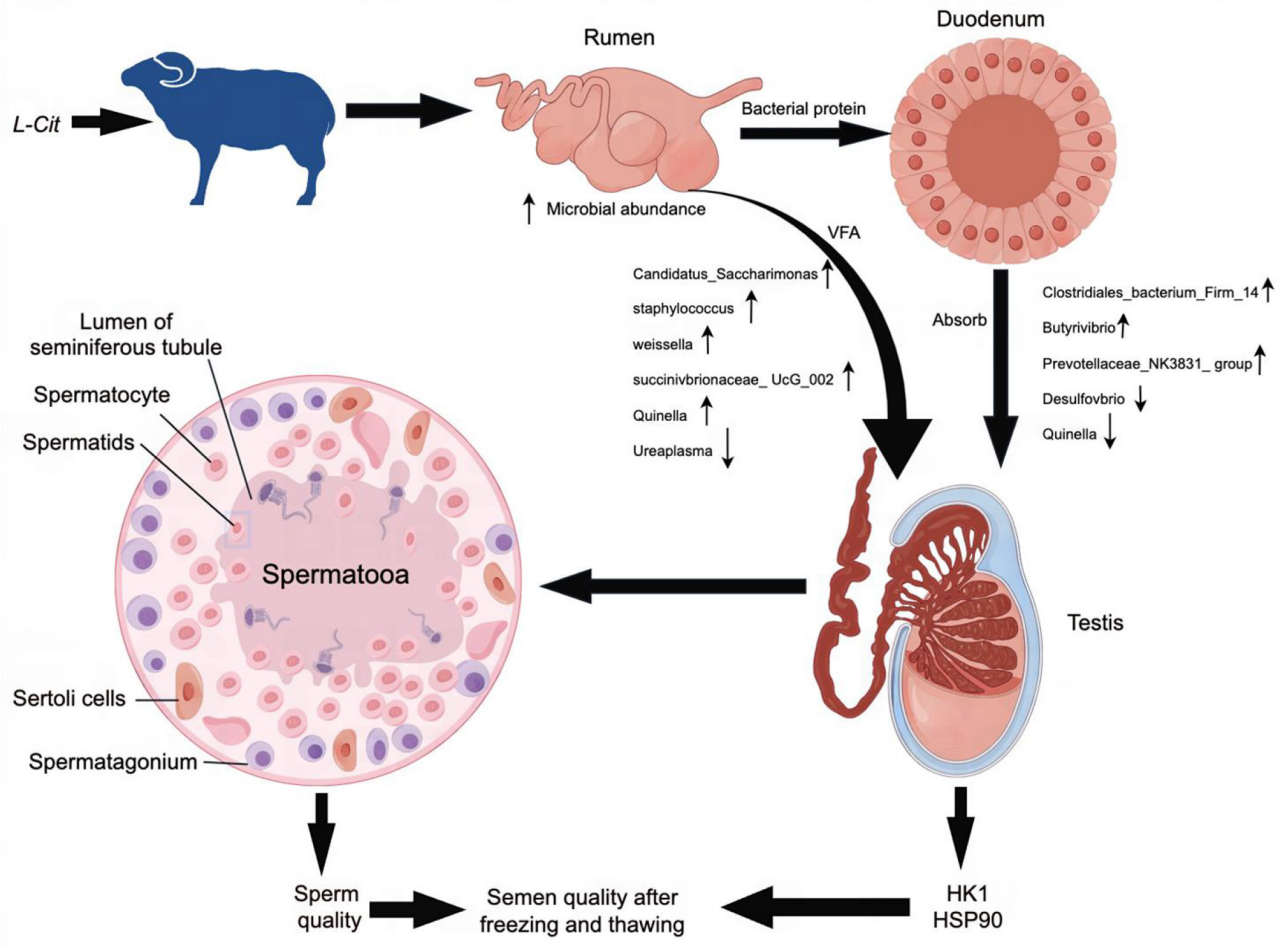
Possible mechanism for the improvement in semen quality by *L*-Cit in this study.

As a precursor of arginine, *L*-Cit may be absorbed and converted into arginine after entering the duodenum to play its physiological role. At present, many scientific studies have shown that there is a close relationship between intestinal microorganisms and the reproductive system. Studies have found that high-fat diet can lead to imbalance of intestinal microbiota in mice, which in turn inhibits spermatogenesis in male mice and significantly reduces sperm motility (Crean and Senior, 2019). Transplantation of fecal microbiota from high-fat-fed mice to normal-fed male mice resulted in a significant increase in the abundance of *Bacteroides* and *Prevotella* in the intestine of normal-fed mice and a decrease in sperm quality (Ding et al., 2020). Liu et al. (2022) found that 250 mg/kg glyphosate had adverse effects on the intestinal microflora of mice, which in turn damaged the testis, reduced sperm motility, and increased sperm deformity rate. Zhang et al. constructed a diet-induced metabolic disorder sheep (Ovis aries) model. Through 16S sequencing analysis, it was found that *Ruminococcaceae-NK4A214-group* was significantly down-regulated in the intestine (Zhang et al., 2022). At the same time, the level of bile acid in sheep decreased sharply, which seriously damaged the absorption process of vitamin A by the host. The disorder of vitamin A metabolism is further transferred to the host testis through blood circulation, which ultimately leads to impaired spermatogenesis in sheep testis. Dai et al. (2015) also confirmed that the increase in the number of microorganisms in the mammalian gut can promote the catabolism of dietary arginine and affect semen quality. The metabolic changes related to cecal microflora in rats showed that *L*-glutamic acid was negatively correlated with *Prevotella* (Han et al., 2019). Although amino acids can alter gut microbiota, they can also maintain host amino acid homeostasis by promoting amino acid digestion and absorption. Pig gut microbiota can promote the synthesis of essential amino acids such as leucine (Leu) required by the host (Torrallardona et al., 2003). These studies have shown that amino acids can regulate gut microbiota and affect the metabolic process of amino acids, thereby improving semen quality. In this experiment, *L*-Cit supplementation changed the microbial abundance in the gastrointestinal tract of sheep, which in turn had a significant effect on semen quality. This finding provides a basis for the application of *L*-Cit in improving the reproductive performance of sheep (Figure 16).

## 5 Conclusions

In conclusion, Under the conditions employed in this study, *L*-Cit supplementary feeding exhibited a significant capacity to increase sperm density among rams, as well as improve TM, PM, and PMI of frozen sperm and the levels of T-AOC, CAT, GSH-Px, and NO within seminal plasma. Furthermore, by conducting correlation analyses on the intestinal flora of rams before and after sperm freezing, we found that *L*-Cit supplementary feeding could improve the abundance of intestinal flora in rams. This, in turn, promotes the development of spermatogonia, spermatocytes, and sperm cells.

## Data availability statement

The data that supports the findings of this study are available from NCBI, accession number PRJNA1101863; publicly accessible at https://www.ncbi.nlm.nih.gov/bioproject/PRJNA1101863/. Other data that supports the findings of this study are available from the corresponding author upon reasonable request.

## Ethics statement

All procedures in this study were approved by the Animal Experiment Ethics Committee of Xinjiang Agricultural University (Protocol Permit Number: 2020032, 7 May 2020; permit number: 2020024, 20 March 2020). The study was conducted in accordance with the local legislation and institutional requirements.

## Author contributions

CF: Data curation, Formal analysis, Writing – original draft. AA: Formal analysis, Writing – original draft. AF: Data curation, Writing – original draft. AD: Data curation, Writing – original draft. XZ: Data curation, Writing – original draft. ZL: Data curation, Writing – original draft. CC: Data curation, Writing – original draft. GZ: Conceptualization, Data curation, Formal analysis, Funding acquisition, Methodology, Resources, Writing – review & editing, Project administration.
